# Evaluation of diet quality of Singaporean mothers and their weaning children

**DOI:** 10.1007/s00394-025-03795-7

**Published:** 2025-09-23

**Authors:** Angel Lin Fung Chi, Allan Lim, Irma Silva Zolezzi, Sharron A. Kuznesof, Chris J. Seal, Iain A. Brownlee

**Affiliations:** 1 Senior Scientific Affairs and Compliance Manager, Nestlé Singapore, 15 A Changi Business Park Central 1, #05-02/03 Eightrium @ Changi Business Park, Singapore, 486035 Singapore; 2 Newcastle Research and Innovation Institute, Devan Nair Building, Singapore, 600201 Singapore; 3 Group Manager, Front End Innovation and Partnerships, Nestlé R&D Centre (Pte) Ltd, 29 Quality Road, Singapore R&D, Singapore, 618802 Singapore; 4 Research and Innovation Unit Lead, Research & Development, SPN, Formerly Nestec S.A., Av. Nestlé 55, 1800 Vevey, Vaud, Switzerland; 5 Senior Lecturer in Food Marketing, Applied Social Sciences - School of Natural and Environmental Sciences, Newcastle University, Newcastle upon Tyne, NE1 7RU UK; 6 Human Nutrition and Exercise Research Centre, Population Health Sciences Institute, Newcastle University, Newcastle upon Tyne, NE2 4HH UK; 7 Faculty of Health and Life Sciences, Northumbria University, Newcastle upon Tyne, NE1 8ST UK

**Keywords:** Dietary pattern, Complementary feeding, Weaning, Food-based guidelines, Nutrient-based guidelines, Diet quality, Healthy eating index, Ethnic differences

## Abstract

**Purpose:**

Studies examining the concordance of the dietary intake of mothers and their weaning children across the food groups are scarce. This study aimed to develop culturally relevant, healthy eating indices to examine the diet quality of mother–child dyads in Singapore.

**Methods:**

488 pairs of mothers with children aged 6–24 months were recruited between August 2017 and December 2018 in this cross-sectional study. Their food frequency questionnaire data were collected for comparison to the national dietary guidelines. ANOVA tests were performed on the diet quality scores of mother–child dyads and comparisons between socioeconomic and demographic characteristics were determined by Mann–Whitney tests with *post hoc* analysis.

**Results:**

Median diet quality score was 57/100 (48.7–65.9) and 65/100 (56.5–72.3) for mothers and children respectively, with intakes of whole grains, vegetables and fruit failing to meet the recommendations. Mothers’ diet quality tended to less frequently align with recommendations than that of the children. The median component score for whole grain was the lowest in mothers and children. Highly educated mothers had higher HEI-SG scores (*p* = 0.022), and significantly higher scores for whole grains (*p* = 0.007), vegetables (*p* = 0.028) and fruit (*p* < 0.001). Children of Chinese descent had higher overall HEI-SG scores than those of non-Chinese ethnicity.

**Conclusions:**

The results highlight common limitations in the dietary habits of Singaporean mothers and their children, which may be used to target the development of future national dietary guidelines.

**Supplementary Information:**

The online version contains supplementary material available at 10.1007/s00394-025-03795-7.

## Introduction

Eating behaviours are believed to emerge in infancy and differ depending on the foods offered during the early period of dietary transition [1]. From approximately six to 24 months, infants start to receive a wider range of food items alongside breast milk and/or formula [2]. Mothers frequently carry a pivotal role in food provisioning [3] that affects the quality of their children’s diet. ‘Diet quality’ often refers to how well an individual’s diet adheres to dietary guidelines. Patterson et al. first published the first diet quality index to assess the overall dietary intake that was related to the risk of chronic diseases in the United States [4]. During the past two decades, the terms ‘healthy eating index’, diet quality index’ and ‘diet quality score’ have emerged in the scientific literature, most often in nutritional epidemiology, to evaluate mainly the dietary habits of adult populations and the efficacy of dietary interventions [5]. The construct of a diet index is comprised of several components, their respective cut-off values, and the criteria for the resultant score to define the optimal diet [6]. Indices have been developed for children and often comprise core food groups, nutrients of concern and diversity of the diet, as well as some eating behaviours [7].

However, there are fewer tools available to consider diet quality during the complementary feeding period. Dietary patterns established during the complementary feeding period appear to track into childhood [8]. Better adherence to complementary feeding guidelines is associated with improved diet at 3 years [9] and later in life [10]. Characterisation of the diet quality of infants and toddlers is scarce, and little is known about how the guidelines are adhered to by the population, and specifically in the case of infants and toddlers, how the parents or caregivers feed the children, with a scarcity of data collected outside of North America and Europe. In Singapore, there have been no national dietary surveys of children below two years of age but recent studies examining the diet of infants and toddlers have recently been published [11–13]. Singapore is also one of only a few countries worldwide that has food-based dietary guidelines that stipulate specific recommendations for feeding solid foods in children younger than 24 months [14, 15].

Therefore, this work aimed to develop a diet quality index based on national dietary guidelines in Singapore and consider the appropriateness of dietary habits in infants and toddlers aged 6–24 months compared with that of their mothers.

## Methods

### Participants

Ethical approval for the study was provided by the School Research Ethics Committee (School of Agriculture, Food and Rural Development), Newcastle University on 14th June 2017 [Ref: 17-LIN-037]. Mothers with children aged 6–24 months were randomly sampled using a computer sample generation method from a commercial database, ‘Nestlé Baby Club’ (NBC) by an independent guardian. NBC was a voluntary membership database offering parenting advice and held details of approximately 26,000 mothers with children of the target age range (i.e., 6–24 months) at the time of sampling (August 2017–December 2018) [16]. A range of pre-defined inclusion and exclusion criteria were used to support screening of the target age group of children. The participating mothers were members of the public with a range of sociodemographic backgrounds within the population.

### Sample size

The calculation for sample size was at least 385 to meet a confidence level of 95% that the real value was within 5% of the surveyed value based on Cochran’s formula [17]. We applied a conservative estimate of the highest realistic attrition rates (20% drop-out, based on the team’s previous wider experience in nutritional research studies) to ensure statistical power was met. As such, a total of 500 participants was initially sought. Singapore is a small country (approximately 726 km^2^) without clear boundaries of socio-economic status [18], thus, further geographical detail was not collected or used in the recruitment process.

### Recruitment process

Potential participants were contacted via phone call. If they met the inclusion criteria and expressed interest in taking part, an email invitation together with a study factsheet would be sent to them. A follow-up call was initiated a few days later by the research team member who then explained the study details to the mothers. The interested mothers who met the criteria were formally recruited. These mothers provided written, informed, itemised, consent for maternal and child data collection activity at the research facility before the start of the study, having been given the opportunity to ask the research team any further questions. They were free to quit at any time without giving a reason for their withdrawal. Due to the nature of the recruitment process, no data were collected from non-respondents and those who declined to participate when they were contacted by the research team.

During study registration, mothers were also asked to report their educational level, household and individual income, employment status, household structure including dwelling type and, number of people in the household. Maternal educational level was divided into four categories (i.e. basic, intermediate, advance I and advance II) ranging from “secondary school leavers” to “postgraduate degree holders”. While data on household income were also collected, the primary analysis focused on educational level, due to Singapore being a high-income country [19].

### Anthropometric data collection

The weight and height of participants were measured at the research facility. Body weight was estimated to the nearest 0.1 kg (with duplicate measure within 0.01 kg) using Seca876 and Seca334 digital scales (Seca, Hamburg, Germany) for mothers and children, respectively. The length of the children to the nearest 0.1 cm was measured using a Rollameter (Seca 417, Hamburg, Germany), while the height of mother was measured to the nearest 0.1 cm using a Stadiometer (Seca217, Hamburg, Germany). Measurements were taken in duplicate and averaged. The mother’s BMI was calculated in kg/m^2^ using the Singaporean standard with the cut- off level at 23 kg/m^2^ for ‘normal’ BMI. Weight status of the child was assessed using weight-for-length using World Health Organisation (WHO) growth charts [20].

### Dietary intake assessment: food frequency questionnaires

The primary dietary intake assessment method used in this study was food frequency questionnaires (FFQs) based on considerations relating to study design, participant burden and the required endpoints for analysis [21]. For the estimation of dietary intake in mothers, a food frequency questionnaire (FFQ-M) adapted from the Singaporean National Nutrition Survey [21, 22] was used (FFQ-M—see Supplementary Material S1). The food frequency questionnaire for children (FFQ-C—see Supplementary Material S2) was adapted from a previously validated tool originally used in a study in China [23] and was seen as the most culturally-relevant, existing questionnaire available, capturing the range of foods used in complementary feeding in Singapore. Nutrient and energy data were estimated based on the compositional database provided by the Health Promotion Board [24].

### Portion size estimation

For the FFQ-C, the original food list was modified to ensure relevance to the local Singaporean diet. The portion of each food was described in a household measurement (e.g., “2 heaped dessert spoons of vegetables”; “½ slice of bread”; “1/3 bowl of rice”). A bank of food images was developed in-house for both FFQs to support improved estimation of portion sizes and used alongside exemplar plates, spoons and other kitchenware. Portion sizes were verified with the research team in the event queries were raised by participants during the FFQs session.

### Design of diet quality scoring systems

Table 1 shows the scoring parameters used for developing the healthy eating indices. Each component was scored from 0 (for lack of adherence) to 10 (for full adherence). The choice to model our diet quality scoring approach with HEI scoring was based on the structural similarity of Singaporean guidelines to those used in the US [11, 25] and the need for a continuous scoring approach for this study, allowing more in-depth exploration of associated factors and correlations. For adequacy components, intakes at the level of the set standard or higher received the maximum number of points. For moderation components, intakes below the recommendation received the maximum number of points. Food and nutrient intake values were energy adjusted and expressed per recommended 1000 kcal. The recommended intake per 1000 kcal of each food group/nutrient was used as the standard against the observed intake per 1000 kcal. The total HEI-SG-M and HEI-SG-C score ranged from 0 to 100 (0 being the lowest and 100 being the maximum score). This approach helps normalise data and limit outliers.

**Table 1:** Proposed scoring structure used in the maternal healthy eating index (HEI-SG-M) and child healthy eating index (HEI-SG-C) for Singaporean mothers (18–50 years old) and children (6–24 months old)

Adequacy components	18–50 years	1–2 years	6–12 months	18–50 years	1–2 years	6–12 months	18–50 years	1–2 years	6–12 months
	Recommended serving^1^ per day			Maximum score of 10 point allocated if serving is equal or more			Minimum score of 0 point allocated if no consumption		
Rice and alternatives^2^	5–7	2–3	1–2	≥ 5	≥ 2	≥ 1	0		
Whole grains^3^	2	½–1	½	≥ 2	≥ ½	≥ ½	0		
Meat and alternatives^4^	2–3	½	½	≥ 2	≥ ½	≥ ½	0		
Dairy and alternatives^5^	½	1½	1½	≥ ½	≥ 1½	≥ 1½	0		
Vegetable^6^	2	½	½	≥ 2	≥ ½	≥ ½	0		
Fruit^7^	2	½–1	½	≥ 2	≥ ½	≥ ½	0		
						Total maximum score = 60 points			

Moderation components	18–50 years	1–2 years	6–12 months	18–50 years	1–2 years	6–12 months	18–50 years	1–2 years	6–12 months
	Recommended Intake (mg per day/% per day)			Maximum score of 10 point allocated if the amount/% is equal or less			Minimum score of 0 point allocated if the amount/% is equal or more		
Sodium^8^ (mg)	≤ 2000	NA	NA	≤ 1000	≤ 975	≤ 390	≥ 2000	≥ 1000	≥ 975
Total fat^9^ (%)	25–30	30–40	30–45	≤ 25	≤ 30	≤ 30	≥ 30	≥ 40	≥ 45
Saturated fat^9^ (%)	≤ 10	≤ 10	≤ 10	≤ 10	≤ 10	≤ 10	≥ 20	≥ 20	≥ 20
Added sugar^9^ (%)	≤ 10	≤ 10	≤ 10	≤ 10	≤ 5	≤ 5	≥ 20	≥ 20	≥ 20
						Total maximum score = 40 points			

^1^Serving per day refers to the daily recommended intake set by the Health Promotion Board, Singapore. ^2^Includes rice, rice porridge, breakfast cereals, noodles, white bread, pasta, toast etc. ^3^Includes whole-grain bread, cereals etc. ^4^Includes all red and white meat, beans and legumes, nuts, tofu, eggs and sea foods. ^5^Includes milk, cheese and yoghurt etc. ^6^Includes green leafy vegetables, root stem, carrots, peppers etc.^7^Includes all kind of fruit. ^8^Dietary guidelines in Singapore limit the salt intake to ≤ 5 g/day for adults, which is equivalent to be ≤ 2000 mg sodium. The maximum score allocated when daily intake is ≤ 1000 mg. Similarly, the maximum score set for children aged 12–24 and 0–12 months old is ≤ 975 mg and ≤ 390 mg per day respectively. ^9^Limits of the percentage amount is based on the total energy required per day

To calculate each component score, the number of servings of each food item consumed by an individual was assessed using the FFQs. For composite dishes (e.g., fried noodles with meat and vegetables), the weight of separate ingredients was estimated by recipe to feed into calculation of multiple component scores (i.e., rice and alternatives, meat and alternatives, and vegetables). The estimated daily intake of each food group or nutrient was then compared to minimum and maximum intake values to derive each component score. The total score was computed as the total of all component scores.

### Statistical analysis

Data were initially evaluated for normality using Statistical Package for Social Science SPSS version 25.0 (IBM SPSS Inc., Chicago, IL, USA). No datapoints were removed within our final analysis. The total diet quality scores for mothers [HEI-SG-M] and children [HEI-SG-C] were presented as the median and interquartile range. Percentages of mothers and children meeting the recommendations for each HEI-SG component were calculated. Pearson correlation was used to determine the association between the BMI and the overall total HEI-SG score of the participants (mother–child dyads). ANOVA (analysis of variance) tests were performed on both HEI-SG total scores. Subsequent analyses were performed to evaluate the characteristics of both mothers and children. Firstly, data were analysed with demographic and other factors by Mann-Whitney tests, with Bonferroni post hoc analyses. A p-value of < 0.05 was considered statistically significant. The data were presented as medians and interquartile range (IQR).

## Results

### Participant details

From 15,348 emails that were sent out, there were a total of 665 follow-up phone calls, with 488 participants recruited. However, two sets of FFQs were void due to missing data resulting in 486 mother–child dyads. Details of the ethnicity representations, anthropometric measurements, demographic characteristics and maternal employment status are tabulated in Table 2.

**Table 2:** Demographic and biological characteristics of participating mothers and children in the study (n = 486)

Mother cohort	18–29 years^1^ (n = 107)	30–50 years^1^ (n = 379)	Child cohort	6–11months (n = 209)	12–24 months (n = 277)
Ethnicity			Ethnicity^2^		
Chinese	82.2% (88)	89.2% (338)	Chinese	83.7% (175)	85.2% (236)
Malay	10.3% (11)	5.5% (21)	Malay	5.3% (11)	4.0% (11)
Indian	4.7% (5)	3.7% (14)	Indian	6.2% (13)	6.8% (19)
Others	2.8% (3)	1.6% (6)	Others	4.8% (10)	4.0% (11)
A household of 2 parents	97.2% (104)	99.2% (376)	Mean age	9.9 ± 1.2	14.6 ± 3.1
Single family	2.8% (3)	0.8% (3)	Male: female	106:103	134:143
Maternal education^3^			Breastfeeding		
Basic	29.9% (32)	12.4% (47)	For the first 6 months: none	160:49	178:99
Intermediate	20.6% (22)	15.3% (58)	Weight (kg) and length (cm)		
Advanced I	44.9% (48)	58.3% (221)	Mean birth weight (kg)	3.1 ± 0.4	3.1 ± 0.4
Advanced II	4.6% (5)	14.0% (53)	Mean body weight (cm)	8.9 ± 1.0	9.9 ± 1.4
			Mean birth length (kg)	49.6 ± 2.4	49.4 ± 2.6
Full-time employment	65.4% (70)	72.3% (274)	Mean body length (cm)	73.1 ± 3.4	77.5 ± 4.3
Weight (kg) and length (cm)			Percentile at birth		
Mean body weight (kg)	58.16 ± 11.55	59.03 ± 12.01	Weight (kg)	38.6 ± 24.4	37.2 ± 25.4
Mean body length (cm)	158.38 ± 5.18	159.58 ± 5.29	Length (cm)	51.3 ± 28.7	50.1 ± 31.9
Mean BMI (kg/m^2^)			Percentile estimated^4^		
(n = 479)^6^	23.20 ± 4.37	23.20 ± 4.62	Weight (kg)	52.0 ± 27.4	50.6 ± 28.8
Underweigt^5^ % (n = 41)	10.7% (11)	8% (30)	Length (cm)	58.9 ± 32.2	47.9 ± 33.1
Normal weight^5^ % (n = 238)	48.5% (50)	50% (188)	BMI-z-score^4^		
Overweight^5^ % (n = 136)	23.3% (24)	29.8% (112)	Underweight % (n = 14)	2.9% (6)	2.9% (8)
Obese^5^ % (n = 64)	17.4% (18)	12.2% (46)	Normal weight % (n = 454)	95.2% (199)	92.1% (255)
			Overweight% (n = 18)	1.9% (4)	5.0% (14)

^1^There is no mean age as mothers were only asked to select their age range. ^2^Children’s ethnicity has previously been standardly defined by paternal ethnic categorisation in Singapore [19]. ^3^Basic: primary, secondary and colleges; intermediate: polytechnic/diploma holders. Advance I: degree holders. Advance II: postgraduate degree holders. ^4^Percentiles and z score calculations were based on WHO growth standard [26]. ^5^BMI calculations were based on HPB ministry of health clinical practice guidelines [27]. ^6^Seven mothers were pregnant (four mothers in the aged range of 18–29 years old and three in the 30–40 years old), making the total 479 valid data in BMI category

### Weight status and BMI

There were no significant differences in mean values of BMI of mothers aged 18–29 years old and 30–49 years old (data not shown). The mean BMI values for both mother groups fell just into the moderate risk category [27]. The weight statuses of mothers and children are shown in Table 2.

### Diet quality scores and daily recommended intake

Table 3 shows that the mean total diet quality scores estimated for mother and child cohorts were 57.5 ± 11.6 and 64.3 ± 11.7, respectively. For mothers, the lowest median component score was found in sodium (0.0 (IQR 0.0–0.0)), followed by the whole grains score (23.1 (6.3–54.7)) and the added sugar score (43.5 (0.0–91.7)). In the child’s cohort, the saturated fat score was the lowest (0.0 (0.0–38.6)), followed by whole grains (6.6 (0–52.9)) and fruit (62.6 (33.2–100)). The percentage of mothers and children meeting the recommendations for each HEI-SG component was different. A large proportion of mothers failed to meet the recommended daily servings for adequate components such as rice and alternatives, whole grains, dairy and alternatives, vegetables and fruit for Singaporeans adults (i.e., n = 163 (34%), n = 47 (10%), n = 165 (34%), n = 74 (15%), n = 127 (26%) respectively). In the child cohort, more than one-third of children met the recommended intake for rice and alternatives, meat and alternatives, and vegetables. The proportion of mothers and children meeting the fruit recommendation was similar. However, the percentage of those meeting the limited intake of sodium and total fat was very different between the mothers and children (i.e., mother’s cohort (n = 0 (0%); n = 61 (13%) respectively and child’s cohort (n = 370 (76%); n = 293 (60%) respectively).

**Table 3:** Median scores for the healthy eating index of mothers (HEI-SG-M) and children (HEI-SG-C), and percentage of participants with maximum and minimum scores for components of the healthy eating index, Singaporean mothers (18–50 years old) and their children (6–24 months old) (n = 486)

Components	Mother cohort				Child cohort			
	Mean	Median (IQR)	Zero score % (n)	Max score % (n)	Mean	Median (IQR)	Zero score % (n)	Max score % (n)
Rice and alternative	86.4 ± 15.5	91.5 (77.5–100)	0.0 (0)	33.5 (163)	76.2 ± 23.6	80.5 (59.7–100)	0.2 (1)	30.2 (147)
Wholegrains	34.1 ± 33	**23.1 (6.3–54.7)**	8.4 (41)	**9.7 (47)**	29.4 ± 37.3	**6.6 (0–52.9)**	43.2 (210)	**11.9 (58)**
Meat and alternative	98.8 ± 5.1	100 (100–100)	0.0 (0)	**90.7(441)**	73.8 ± 30.8	89.3 (52.5–100)	2.7 (13)	39.1 (190)
Dairy and alternative	63.6 ± 34.5	66.8 (34.3–100)	3.1 (15)	34 (165)	71 ± 18.1	71 (58.7–83.5)	0.0 (0)	10.3 (50)
Vegetables	62.8 ± 25.4	60.8 (41.9–84.2)	0.0 (0)	15.2 (74)	75.3 ± 29.1	89 (52.9–100)	1.4 (7)	44.9 (218)
Fruit	63.5 ± 30.6	64.1 (37.7–100)	0.2 (1)	26.1 (127)	61.6 ± 32.9	62.6 (33.2–100)	2.3 (11)	27.6 (134)
Sodium	0.2 ± 3.1	0(0.0–0.0)	99.2 (482)	**0.0 (0)**	76.1 ± 42.7	100 (100–100)	23.9 (116)	**76.1 (370)**
Total fat	47.3 ± 34.5	45.9 (15.9–77.1)	14.8 (72)	**12.6 (61)**	67 ± 44.7	100 (0–100)	28.2 (137)	**60.3 (293)**
Saturated fat	71.3 ± 18.1	71.4 (59.1–84)	0.0 (0)	8.6 (42)	20.1 ± 31.4	0 (0–38.6)	63.0 (306)	0.0 (0)
Added sugar	46.7 ± 40.2	43.5 (0–91.7)	29.8 (145)	20.4 (99)	92.9 ± 12.9	97.2 (92.9–99.2)	0.6 (3)	9.3 (45)
Overall (total)	**57.5 ± 11.6**	**57.5 ± 11.6**			**64.3 ± 11.7**	**64.3 ± 11.7**		

### Correlations and ANOVA tests

Results of Pearson’s analyses did not show correlations between the total HEI-SG score and BMI status for both mother (*r* = − 0.082 *p* = 0.072) and children (*r* = 0.015; *p* = 0.734) groups respectively. The one-way ANOVA test indicated a significant difference in the mother’s HEI-SG total score between the four education groups (*p* = 0.014), but no significance was shown in the four BMI categories. However, when combining the ‘overweight’ and ‘obese’ into one category, the HEI-SG total score showed a statistical difference between the three BMI categories (*p* = 0.042) (see Table 4). In the child cohort, ethnicity (*p* < 0.0001) and gender (*p* = 0.004) of children showed significant differences in HEI-SG total scores between the groups.

**Table 4:** Evaluation of the HEI-SG total score by demographic and biological characteristics

Mother cohort				Child cohort			
Variable	n	HEI-SG-M total score [Mean ± SD]	*p*	Variable	n	HEI-SG-C total score [Mean ± SD]	*p*
Age				Age			
18–29 year	107	57.3 ± 9.1	0.364	6–11 months	209	60.7 ± 11.3	0.364
30–50 year	379	58.3 ± 9.8		12–24 months	277	65.4 ± 10.4	
Ethnicity				Ethnicity			
Chinese	426	58.0 ± 9.6	0.068	Chinese	411	63.9 ± 10.7	**0.000**
Indian	19	63.4 ± 10.5		Indian	21	56.3 ± 13.9	
Malay	32	58.1 ± 8.4		Malay	31	58.4 ± 11.0	
Others	9	57.5 ± 11.0		Others	23	66.8 ± 10.7	
Education levels				Gender			
Basic	80	56.4 ± 9.3	**0.014**	Female	246	61.9 ± 10.8	**0.004**
Intermediate	80	58.9 ± 9.9		Male	240	64.8 ± 11.2	
Advance I	269	57.6 ± 9.8					
Advance II	57	61.4 ± 10.0		Birth order			
				1 st Child	283	63.5 ± 10.9	0.921
Employment status				2nd Child	142	63.4 ± 11.0	
Full time	344	57.8 ± 9.6	0.324	2 or more siblings	61	62.8 ± 12.1	
Homemaker	96	59.3 ± 9.8					
Part time	37	57.6 ± 10.1					
Others	9						
				Z score category			
				Underweight	14	62.6 ± 8.9	0.95
Weight status^1^				Normal	454	64.4 ± 11.0	
Underweight	41	61.5 ± 10.3	**0.042**	Overweight	18	62.6 ± 13.4	
Normal	238	58.4 ± 9.5					
Overweight + Obese	200	57.1 ± 9.7					

^1^Seven mothers were pregnant at the time of data collection and their data were excluded from the analysis. Total sample size for evaluating the HEI-SG-M store and maternal weight status was 479. ANOVA tests were performed on both HEI-SG total scores

### Demographic and other factors affecting the HEI-SG score

Tables 5, 6 and 7 show the HEI- SG scores of both cohorts by their demographic and biological characteristics. No significant difference in the overall total HEI-SG score between the two age groups of mothers, however, the older mother group showed statistically higher component scores for vegetables (*p* = 0.030) and fruit (*p* = 0.003), but lower for rice and alternatives (*p* = 0.043). Of note, older children (aged 12–24 months) had statistically higher (*p* < 0.001) overall total HEI scores than younger children (aged 6–11 months), contributed mainly by the components of meat and alternatives, vegetables, and fruit (see Table 5).

**Table 5:** Evaluation of healthy eating index (HEI-SG) by age group of mothers and children (n = 486)

Component	Mother cohort		*p*	Child cohort		*p*
	18–29 year (n = 107)	30–49 year (n = 379)		6–11 month (n = 209)	12–24 month (n = 277)	
	Median (IQR)			Median (IQR)		
Rice and alternatives	95.5	89.9	**0.043**	98.1	72.3	**< 0.001**
	(78.9–100)	(76–100)		(72.8–100)	(53.3–90.6)	
Whole grains	14.9	24.9	0.274	1.7	12.8	**0.007**
	(4.6–56)	(7–54.4)		(0–41.0)	(0–66.2)	
Meat and alternatives	100	100	0.732	64.3	100	**< 0.001**
	(100–100)	(100–100)		(38.1–95.8)	(69.0–100)	
Dairy and alternatives	63.4	67.9	0.513	69.4	71.7	0.078
	(30–100)	(35.1–100)		(58.6–79.6)	(59.2–87.7)	
Vegetables	56.9	63.1	**0.030**	75.3	100	**0.003**
	(36.5–75.6)	(42.8–86.9)		(46.3–100)	(60.1–100)	
Fruit	51.1	67.5	**0.003**	51.0	74.3	**< 0.001**
	(28.1–83.1)	(40.4–100)		(28.4–83.8)	(39.8–100)	
Sodium	0	0	0.174	100	100	**< 0.001**
	(0.0–0.0)	(0.0–0.0)		(0–100)	(100–100)	
Total fat	50.7	43.8	0.300	100	100	**< 0.001**
	(13.1–86.1)	(16.0–75.1)		(76.8–100)	(0–100)	
Saturated fat	72.4	71.4	0.381	0	0	0.578
	(59.1–87.7)	(59.1–82.3)		(0–38.1)	(0–38.4)	
Added sugar	49.7	42.8	0.915	97.7	96.4	**< 0.001**
	(0.0–87.3)	(0.0–93.3)		(95.2–99.3)	(89.3–99.0)	
Overall (total)	55.0	57.6	0.326	61.0	66.2	**< 0.001**
	(48.7–65.5)	(48.7–66.2)		(52.9–70.2)	(59.7–72.9)	

**Table 6:** Evaluation of healthy eating index (HEI-SG) by ethnicity of mothers and children

	Mother cohort						Child cohort				
	Chinese (n = 426)	Malay (n = 32)	Indian (n = 19)	Others (n = 9)			Chinese (n = 411)	Malay (n = 22)	Indian (n = 32)	Others (n = 21)	
	Median (IQR)				*P*		Median (IQR)				*p*
Rice and alternative	91.2	91.9	98.5	93.2			80	92	86.9	71.4	
	(76.9–100)	(78.8–100)	(80.0–100)	(86.5–100)	0.539		(58.9–100)	(65.9–100)	(70.5–100)	(58.9–100)	0.325
Whole grains	22.8	19.1	32.4	21.5			7.2	0	15.4	4.4	
	(6.7–55.7)	(4.5–39.1)	(18.9–55.2)	(1.7–49.3)	0.546		(0–55.5)	(0–24.4)	(0–52.1)	(0–28.3)	0.325
Meat and alternative	100.0 ^a^	100.0 ^a^	100.0 ^b^	100.0 ^ab^			90.4	78.8	61.4	77.5	
	(100–100)	(100–100)	(88.2–100)	(100–100)	**< 0.001**		(52.9–100)	(53.7–100)	(36.1–100)	(55.1–95.8)	0.331
Dairy and alternative	61.0	88.1	100.0	100.0			71.2	74.3	66.1	72.3	
	(33.2–100)	(50.8–100)	(51.8–100)	(49.2–100)	**0.015**		(58.7–84.4)	(56.7–79.2)	(53.9–78.6)	(62.3–76.4)	0.657
Vegetables	62.7 ^a^	49.0 ^b^	56.7 ^ab^	50.2 ^ab^			89.0 ^b^	63.7 ^b^	100 ^a^	100 ^ab^	
	(43.9–85.3)	(32.6–69)	(45.2–76.7)	(37.1–100)	**0.044**		(55–100)	(31.3–100)	(38.0–100)	(43.9–100)	**0.049**
Fruit	64.6	48.2	81.5	57.3			61.2	61.1	91.2	100	
	(38.7–100)	(26.2–100)	(50.6–100)	(29.5–100)	0.512		(33.4–100)	(28.4–87.3)	(43.7–100)	(49.8–100)	0.222
Sodium	0	0	0	0			100 ^a^	100 ^b^	100 ^b^	100 ^ab^	
	(0–0)	(0–0)	(0–0)	(0–0)	0.500		(100–100)	(0–100)	(0–100)	(0–100)	**< 0.001**
Total fat	45.0 ^b^	59.8 ^ab^	88.2 ^a^	36.2 ^ab^			100	100	72.9	100	
	(15.0–75.0)	(15.5–77.7)	(34.2–100)	(34.1–66.2)	**0.023**		(0–100)	(60.8–100)	(0–100)	(100–100)	0.222
Saturated fat	71.6	66.5	88.5	59.9			0	0	0	29.7	
	(59.6–83.4)	(55.4–80.6)	(58.1–96.9)	(49.5–83)	0.232		(0–37.6)	(0–57.8)	(0–6.8)	(0–57.6)	**0.043**
Added sugar	46.6 ^b^	0.0 ^ab^	62.9 ^a^	0.0 ^b^			97.6 ^a^	92.7 ^b^	95.7 ^ab^	96.3 ^ab^	
	(0–93.3)	(0–75.8)	(10.4–100)	(0–26.8)	**0.032**		(93.6–99.3)	(83.5–96.5)	(88.9–97.9)	(89.0–99.3)	**< 0.001**
Overall (total)	56.8^b^	54.6 ^b^	62.8 ^a^	51.1 ^ab^			65.0 ^a^	59.1 ^b^	59.3 ^b^	68.0 ^ab^	
	(48.8–66.2)	(47.2–60.9)	(58.4–70.9)	(45.5–60.1)	**0.044**		(57.2–72.3)	(55.0–65.0)	(48.8–69)	(61.2–74.9)	**0.009**

Sub-groups with different superscripts (^a^/^b^/^c^/^d^) in a single row are statistically different (*p* < 0.05) by Mann-Whitney tests with Bonferroni’s post hoc test

**Table 7:** Evaluation of healthy eating index (HEI-SG) by weight status of mothers (n = 479) and children (n = 486)

Component	Mother cohort (n = 479)					Child cohort (n = 486)			
	Underweight^1^ (n = 41)	Normal^2^ (n = 238)	Overweight^3^ (n = 136)	Obese^4^ (n = 64)		Underweight^1^ (n = 14)	Normal^2^ (n = 454)	Overweight^3^ (n = 18)	
	Median (IQR)				*p*	Median (IQR)			*p*
Rice and alternative	94.3	93.5	89.7	85.3		86.3	80.6	71.8	
	(86.1–100)	(78.9–100)	(77.4–100)	(72.4–100)	**0.042**	(66.5–100)	(59.9–100)	(55.0–92.2)	0.255
Whole grains	32.3	25.1	21.6	18.7		0	7.0	0	
	(8.8–88.3)	(5.6–55.2)	(6.3–47.9)	(5–49.7)	0.271	(0–83.0)	(0–53.2)	(0–28.3)	0.473
Meat and alternative	100	100	100	100		57.6	89.3	100	
	(100–100)	(100–100)	(100–100)	(100–100)	0.396	(30.6–100)	(53.7–100)	(36.5–100)	0.228
Dairy and alternative	51.2	69.5	72.7	68.9		67.5	71.0	68.8	
	(25.8–94.3)	(33.4–100)	(37.4–100)	(38.5–100)	0.335	(53.5–83.5)	(58.8–83.3)	(55–98.6)	0.930
Vegetables	66.0	63.0	58.6	59.3		81.3	89.0	91.4	
	(44.3–90.2)	(41.7–85.4)	(44.1–84)	(38.6–77)	0.764	(42.0–100)	(53.6–100)	(46.1–100)	0.888
Fruit	61.9	63.5	67.3	64.1		57.6	63.5	58.1	
	(37.5–100)	(37.2–100)	(34.4–97.5)	(42.3–100)	0.956	(40.7–75.1)	(31.2–100)	(35.2–100)	0.728
Sodium	0	0	0	0		100	100	100	
	(0.0–0.0)	(0.0–0.0)	(0.0–0.0)	(0.0–0.0)	0.605	(0–100)	(100–100)	(100–100)	0.709
Total fat	65.2	47.5	39.4	37.3		100	100	91.0	
	(41.7–98)	(16.5–79)	(6.2–71.1)	(14.7–88.8)	**0.029**	(41.2–100)	(0–100)	(15.9–100)	0.575
Saturated fat	72.9	71.6	69.7	71.4		0	0	0	
	(64.5–94.3)	(59.4–83.6)	(59.1–80.7)	(57.3–87.5)	0.125	(0–39.4)	(0–38.4)	(0–12.5)	0.893
Added sugar	51.8	45.3	40.4	35.5		94.5	97.2	98.5	
	(0–86.7)	(0–95.8)	(0–89.7)	(0–73.9)	0.469	(86–98.1)	(93.1–99.2)	(97.7–99.8)	0.056
Overall (total)	60.4	57.7	56.0	54.4		65.3	64.4	62.3	
	(51.1–71.6)	(48.9–66.3)	(48.5–64.4)	(48.4–64.4)	0.110	(57.2–69.5)	(56.5–72.3)	(56.7–72.6)	0.908

*Seven mothers were pregnant at the time of data collection and their data were excluded from the analysis. ^1^Underweight: BMI below 18.5 kg/m^2^; ^2^Normal weight: BMI 18.5–22.9 kg/m^2^; ^3^Overweight: BMI between 23–29.9 kg/m^2^; ^4^ Obese BMI > 30.0 kg/m^2^. Superscripts (a/b) for BMI category group are statistically different (*p* < 0.05) by Mann–Whitney tests with Bonferroni’s post hoc test

Table 6 shows that the overall total HEI- SG-M score was affected by ethnicity, with those of Indian ethnic origin tending to have a higher total overall HEI-SG score (62.8 (58.4–70.9) compared to Chinese (56.8 (48.8–66.2)) and Malay (54.6 (47.2–60.9)) mothers (*p* = 0.044). A comparison of Chinese mothers (n = 426) and all other ethnic origin groups (n = 60) suggested that Chinese mothers scored higher for the meat and alternatives component (*p* < 0.001, 100.0 (100–100)) and vegetables (*p* = 0.014, 62.7 (43.9–85.3) (data not shown). The ethnicity of the children had a significant effect on the total overall HEI-SG score (*p* = 0.009). A *post hoc* analyse with repeated Mann–Whitney tests for pairing the groups showed that Chinese children had a statistically higher total overall HEI-SG score (65.0 (57.2–72.3)) than Singaporean Indian (59.3 (48.8–69)) and Malay (59.1 (55.0–65.0)) children which were not different from each other. It was also shown that Malay children scored statistically lower for the components: Sodium (*p* < 0.001) and Added Sugar (*p* < 0.001).

Table 7 shows that there were no statistically significant differences in the total overall diet quality across the different BMI categories. Obese mothers had a significantly lower score for rice and alternatives (*p* = 0.042) and total fat components (*p* = 0.029). Further analysis comparing a combined obese and overweight group against normal and underweight groups, showed that significantly lower median scores for ‘rice and alternatives’ (*p* = 0.017) and ‘total fat (*p* = 0.013)’ were observed between the obese and overweight group and underweight group (data not shown). In the child cohort, no significant difference in the HEI-SG scores were observed across the different weight categories (*p* > 0.05), though overweight children appeared to score the lowest.

Table 8 shows that highly educated mothers had a significantly higher total overall HEI-SG-M score (62.5 (50.2–71.6) in the Advanced II group versus 55.1 (48.1–61.1) in the Basic education group; *p* = 0.022y), contributing mainly by ‘whole grains’ (*p* = 0.007), ‘meat and alternatives’ (*p* = 0.017), ‘vegetables’ (*p* = 0.028) and ‘fruit’ (*p* < 0.001). *Post hoc* analyses with repeated Mann–Whitney tests for pairing the groups, showed that between mothers with Advanced II and Basic education, the Advanced II educated mothers scored significantly higher for the components whole grains (*p* = 0.08), vegetables (*p* = 0.003) and fruit (*p* < 0.001).

**Table 8:** Evaluation of HEI-SG-M by maternal education

Component	Basic ^1^ (n = 80)	Intermediate^2^ (n = 80)	Advanced I ^3^ (n = 269)	Advanced II^4^ (n = 57)	*p*
	Median (IQR)				
Rice and alternative	91.8 (77.5–100)	97.0 (84.9–100)	89.4 (76.4–100)	94.8 (72.9–100)	0.171
Whole grains	12.1 ^a^ (3.3–33.6)	38.1 ^b^ (6.5–76.5)	24.1 ^ab^ (7–51.6)	24.9 ^b^ (9.9–70.2)	**0.007**
Meat and alterative	100 (100–100)	100 (100–100)	100 (100–100)	100 (100–100)	**0.017**
Dairy and alternative	64.4 (34.0–100)	60.0 (33.8–100)	69.5 (34.4–100)	67.6 (42.6–100)	0.948
Vegetables	52.9 ^a^ (37.2–78.9)	59.3 ^a^ (42.5–84.8)	60.7 ^ab^ (41.5–84)	72.3 ^b^ (53.5–93.2)	**0.028**
Fruit	53.9 ^a^ (31.1–97.4)	55.7 ^a^ (26.3–87.5)	61.9 ^a^ (39.1–96.0)	98.9 ^b^ (65–100)	**< 0.001**
Sodium	0 (0.0–0.0)	0 (0.0–0.0)	0 (0.0–0.0)	0 (0.0–0.0)	0.255
Total fat	54.9 (15.1–83.9)	49.0 (17.5–86.3)	41.0 (15.0–71.8)	48.6 (24.4–72.7)	0.373
Saturated fat	74.6 (59.1–84.5)	71.7 (56.3–89.4)	69.9 (59.1–81.4)	73.4 (63.4–88.9)	0.261
Added sugar	35.1 (0–77.1)	41.3 (0–83.8)	43.7(0–95.4)	58.0 (0–90.9)	0.598
Overall (total)	55.1^a^ (48.1–61.1)	57.4 ^ab^ (48.8–67.1)	57.0^a^ (48.5–65.6)	62.5 ^b^ (50.2–71.6)	**0.022**

^1^With primary, secondary and college education; ^2^Polytechnic or diploma holders; ^3^ Degree holders; ^4^Postgraduate degree holders. Sub-groups with different superscripts (a/b) for education attainment in a single row are statistically different (*p* < 0.05) by Mann-Whitney tests with Bonferroni’s post hoc test; ^5^ working hours ≥ 30 h a week; ^6^working hours < 30 h a week; ^7^ unemployed- look for a full-time employment; ^8^ unemployed- stay home mothers

Table 9 shows that boys had a higher overall HEI-SG score than girls (65.6 (57.2–73.5) versus 63.5 (55.4–70.6), *p* = 0.024). The components contributing most to this difference appeared to be rice and alternatives (*p* = 0.041), dairy and alternatives (*p* = 0.004) and fruit (*p* = 0.017).

**Table 9:** Evaluation the healthy eating index (HEI-SG-C) by gender of the children (n = 486)

Component	Female (n = 246)	Male (n = 240)	*P*
	Median (IQR)		
Rice and alternatives	76.8 (58.9–100)	84.9 (61.1–100)	**0.041**
Whole grains	7.1 (0–53.5)	6.4 (0–51.0)	0.713
Meat and alternatives	88.6 (49.8–100)	90.6 (53.8–100)	0.413
Dairy and alternatives	69.1 (58.0–79.6)	73.6 (61.2–87.4)	**0.004**
Vegetables	82.5 (47.9–100)	97.4 (54.8–100)	0.113
Fruit	57.6 (31.2–89.0)	69.2 (35.4–100)	**0.017**
Sodium	100 (0–100)	100 (100–100)	0.362
Total fat	100 (0–100)	100 (0–100)	0.393
Saturated fat	0 (0–39.9)	0 (0–37.8)	0.855
Added sugar	97 (93.3–99.2)	97.7 (92.4–99.2)	0.435
Overall (total)	63.5 (55.4–70.6)	65.6 (57.2–73.5)	**0.024**

## Discussion

This study adapted an existing and well-utilised diet scoring system (HEI-2015) [28] to develop two sets of culturally relevant, healthy eating indices (i.e., HEI-SG-M and HEI-SG-C), for assessing the diet quality of Singaporean mothers and their children. The HEI-SG-M score highlighted key areas that could be targeted to improve the mothers’ diet to better align with the national food- and nutrient-based guidelines set by the Singapore Health Promotion Board (HPB). The mean overall total HEI-SG-M score was estimated as 57.5 ± 11.6 (median, 57.0; IQR = 48.7–65.9), consistent within the range of the scores observed in another study [29]. For the HEI-SG-C score, the studies [11, 30, 31] available do not have the same scoring criteria, which makes direct comparison difficult. One recent study has highlighted generally high adherence (mean = 80%) to a discrete, behaviour-based scoring system (the Complementary Feeding Utility index) in a sample of UK children [32].

The component in which mothers mostly scored the highest was ‘meat and alternatives.’ In contrast, 99% of mothers had the minimum component score ‘zero’ for sodium, with the median score at 0 (IQR 0.0–0.0), representing a mean intake of salt at 6.5 g/d which exceeded the limit set by the HPB by 1.5 g. A substantial number of mothers also failed to restrict their total fat (15%) and added sugar (30%) intakes. These findings are in broad agreement with previous, national survey data from Singapore [33]. Previous work has suggested major sources of dietary sodium to be condiments and elective addition of table salt [34]. Out-of-home food consumption has been suggested to be a major driver for excess salt intake in Singaporean adults [35]. In contrast, most children (> 90%) scored 100 for sodium, suggesting that their sodium consumption adhered to the guidelines. Of note, older children (> 12 months) scored slightly lower for ‘added sugar’ when compared with children aged below 12 months. Such a downward trending score was probably related to higher exposure to out-of-home foods, table foods and convenient foods along the transition from toddlerhood to childhood. This echoes the survey report from the Singapore Longitudinal Early Development Study (SG LEADS), indicating that 30% of the studied participants aged 2–6 years often consumed sugar-sweetened beverages (≥ 3 times per week) [36]. This insight into the consumption trend of sugary drinks in preschool children supports the observed increasing trend of consumption for this component in this study cohort. When, and how such sugary foods are introduced into the toddlers’ and the pre-schoolers’ diet is unclear and warrants further study.

For the total fat component, mothers scored 47.3 ± 34.5 (mean) and (45.9 (15.9–77.1) (median IQR) respectively, suggesting that a considerable proportion of mothers may have exceeded the fat energy percentage of 30%. This tallied with the median total fat energy percentage (35%) observed. In the child cohort, fat energy percentage at 35% and 10% was estimated for total fat and saturated fat respectively. Similar results were previously reported on children aged 1–3 years in Malaysia [37]. The HEI-SG score estimated for whole grains was very low in both mothers and children, with only approximately 10% and 12% meeting the daily recommended intake respectively. In the Singapore National Nutrition Survey, 27% of adults reported consuming only 1 ‘whole grains’ portion a day [22]. Such low intake could be associated with its poor low palatability and difficulty in identifying whole-grain products [38]. Dietary intake studies targeting mother’s consumption of whole grains are limited. To the author’s knowledge, only one recent study [39] investigating whole grains consumption in pregnant women in Singapore is available. This study reported an overall median intake of 0 (IQR = 0–9.0) g/day, well below the recommended intake of approximately 60 g per day. In the child cohort, almost half of the children had never consumed whole grains, consistent with the GUSTO prospective cohort study, examining 561 Singaporean children aged 12–24 months old [11].

The current study did not find an association between diet quality and BMI in either mothers or children. These findings do not align with those of previous work where higher BMI is negatively associated with the diet quality [40–42]. Most previous studies have been carried out in populations with high prevalence of obesity. Within the current study, there were a limited number of mothers within the obese category, despite the use of lower BMI category cut-off thresholds. Recent reports suggest national prevalence of obesity in Singapore to be approximately 20% in the adult population [43]. The lack of statistical difference could be due to the small sample size of the obese mothers within the current study, which may be an artefact of the sampling strategy (discussed below) or interest in participating in nutritional studies.

Advancing age has been related to higher diet quality [44]. In the current study, older mothers tended to have higher median diet quality scores, contributed mainly by the vegetables (*p* = 0.03) and fruit (*p* = 0.003) components. The Singapore National Nutrition Survey [22] reported that only 11.2% of adults in Singapore met the recommended 2 servings for both vegetables and fruit and the young adults (aged 18–30 years old) tended to consume fewer vegetables and fruit than the older adults. The present work aligns with this general understanding, showing that 15% and 26% of mothers adhered to the respective recommended servings, and fewer vegetables and fruit were consumed by the younger mother group (aged 18–29 years old). Similarly, older children in this cohort had a significantly higher overall total HEI-SG score.

The main contributors to this higher score were meat and alternatives (*p* = < 0.01), vegetables (*p* = 0.03) and fruit (*p* = < 0.01). A possible reason for this finding could be related to texture preference increasing with age [45]. Moreover, it may partially be due to delayed solid food introduction, since infant formulas in the current study were a major contributor to the foods fed to the younger children group (6–11 months old). Overall, only 36% of children met the recommended servings of vegetables and fruit. Chen et al. also observed a similar low intake of vegetables/fruit in a previous cross-section of Singaporean children [11]. To promote vegetable and fruit consumption, it has been suggested that toddlers should be exposed to a variety of tastes and flavours which helps to increase acceptance of vegetables and fruit [46].

Indian mothers scored the highest HEI-SG score in this study (*p* = 0.044); however, the sample size of minority groups in the study cohort was sub-optimal (n = 19 Indian; n = 32 Malays and n = 9 Others), so caution is needed to interpret such results to avoid over-representation. However, Malay mothers scored the lowest median scores for vegetables and fruit, which is consistent with previous findings [47–50] which suggests that Malay adults tended to have less healthy diets than other ethnic groups. Vegetable and fruit intake, like other aspects of a healthy diet, can be considered a function of socioeconomic status and ethnicity [51, 52]. The small sample size in this cohort limited the potential to explore influence of ethnicity on diet. Ethnic differences in food provisioning habits [53] and the quality of children’s diet have been reported [54], particularly in children of Malay origin [55]. The lower-quality diet found in Malay children was associated with cultural food preferences whereby Malay children were more often offered juices and sweetened drinks when compared with children of Chinese origin [55]. Chinese children in this cohort had statistically higher total overall HEI-SG scores than children of Indian and Malay origin. This aligns with the study findings [56] conducted in older Singaporean children, suggesting that poor dietary habits experienced in early life, even during the complementary feeding period may translate into poor diets throughout childhood and into later life.

The findings revealed that the BMI status of the participants did not affect the HEI-SG scores, contradicting the current understanding which shows that higher BMI is negatively associated with the quality of diet, and adults with a BMI > 40 kg/m^2^ had the lowest diet quality [40, 41]. The lack of statistical difference across the BMI categories could be due to the limited size of the sample in the overweight and obese groups in the respective cohort.

Ross et al. observed that mothers who were degree holders had significantly higher whole-grain intake than secondary school leavers [39]. Mothers who received higher education apparently may be more willing to accept whole grains. Higher-educated mothers in the current study not only consumed more whole grains but also more vegetables and fruit, suggesting education may have a positive influence on the consumption of these food items. Education has been shown to positively influence the vegetables and fruit intake as well as the types of vegetables and fruit that were chosen [57]. Other studies have also shown that education was strongly associated with vegetable and fruit intake [58–60] affecting the overall diet quality. Education level, which is a strong socioeconomic indicator, influences food intake because dietary knowledge and healthfulness work hand in hand while making food choice decisions [61]. Feeding represents the earliest form of parental influence and food provisioning practices differ by gender [62]. Boys in this study had higher component scores for rice and alternatives and dairy and alternatives. Boys tend to require higher energy and more protein than girls due to differences in their physiological makeup. Rice is the main source of energy and dairy is a protein-rich food for toddlers, this may explain the higher intake of these food items observed.

### Strengths and limitations

One of the strengths of this study is that the approach undertaken to construct the healthy eating indices accounted for the anticipated differences by age, gender, and physical activity level across this cross-sectional study. To further increase the precision of data collection, a few measures including (i) dietary intake was energy-adjusted; (ii) added sugar was assessed based on a published systematic approach [63]; (iii) on-site measurements on the height and weight; (iv) all the questionnaires were thoroughly vetted through by the same research member upon completion. However, reporting bias using the food frequency questionnaire was inevitable, with the potential for over-reporting of food perceived as ‘healthy’ [64] and the total energy intakes [65]. Compositional data and incomplete nutrient information for some local Asia foods contributed to the limitation of dietary nutrient and serving estimates.

While the overall sample size was appropriately large in terms of statistical power, there appear to be some limitations in representativeness. Further multivariate analysis would be helpful to consider the relationship between potentially confounding factors here (e.g. age and employment status) but this has not been carried out to date. Specifically, a small sample size of Malay and Indian descendants in this study could have limited the potential to identify the ethnical differences in diet quality. As such, findings should be interpreted cautiously. Our route of participant recruitment (through the Nestlé Baby Club) may not have been truly representative of the wider population of Singaporean mothers but represented the most appropriate strategy available to the research team. Nonetheless, the current work highlights the potential for relevant approaches to take in future national screening programmes.

Finally, the limitations of self-reported dietary data, including FFQs are widely acknowledged [66–69]. We have attempted the mitigate these issues here by using culturally-relevant data collection tools. As per the original HEI, normalisation of nutrient intake data and food frequency against energy intake (per 1000 kcal) helps to limit issues with under- and over-reporting [25].

## Conclusions

The present study evaluated the adherence of mothers and their children to national dietary recommendations. This has identified key areas of sub-optimal intake in the dietary habits of mothers and weaning children. The findings provide a framework for the development of future intervention programmes to foster more positive eating habits in mothers and their children. In addition, a well-designed longitudinal study with a larger number of ethnic groups should be considered in the future.

## Supplementary Information

Below is the link to the electronic supplementary material.

Supplementary Material 1
