# Supplementary material for: Evaluation of diet quality of Singaporean mothers and their weaning children
Source: Eur J Nutr. 2025 Sep 23;64(7):282. doi: 10.1007/s00394-025-03795-7 (PMC12457524; doi:10.1007/s00394-025-03795-7)
Supplement: Supplementary file 1 — Supplementary Material 1 [file 394_2025_3795_MOESM1_ESM.docx]

**Appendix 1 Food Frequency Questionnaire for the Mothers (FFQ-M)**


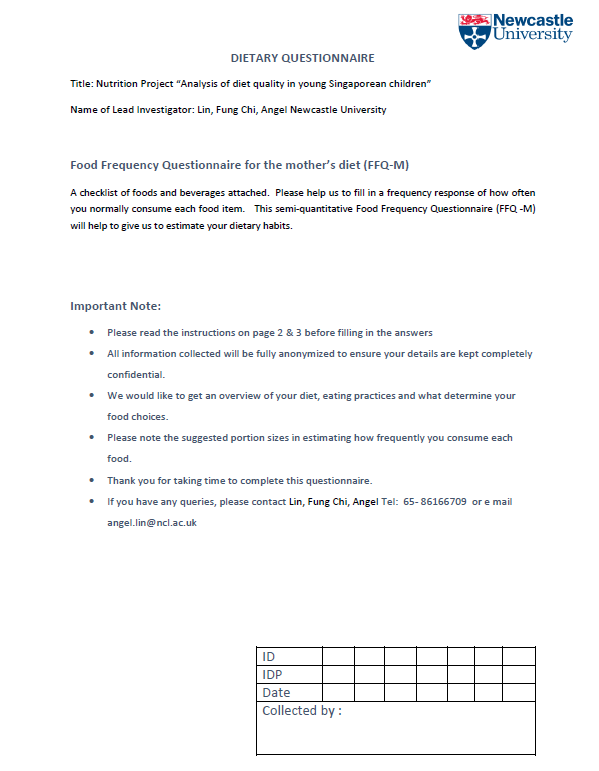

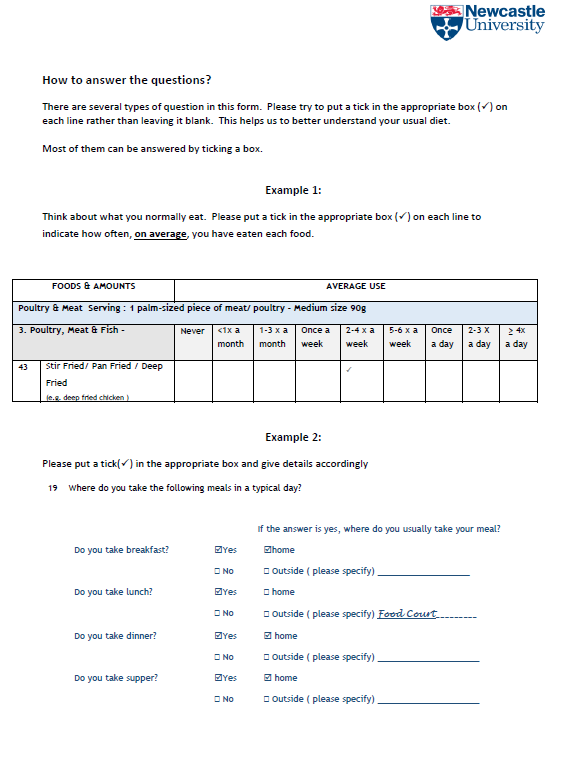


**Appendix 1 Food Frequency Questionnaire for the Mothers (FFQ-M)**


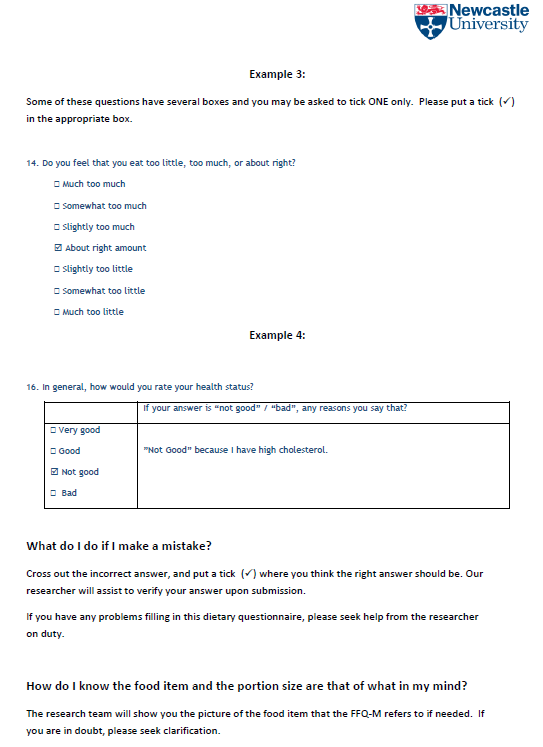

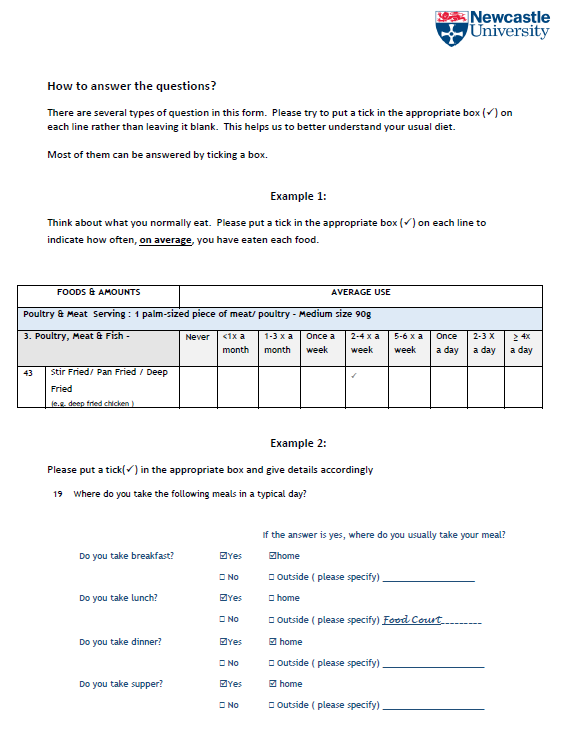


**Appendix 1 Food Frequency Questionnaire for the Mothers (FFQ-M)**


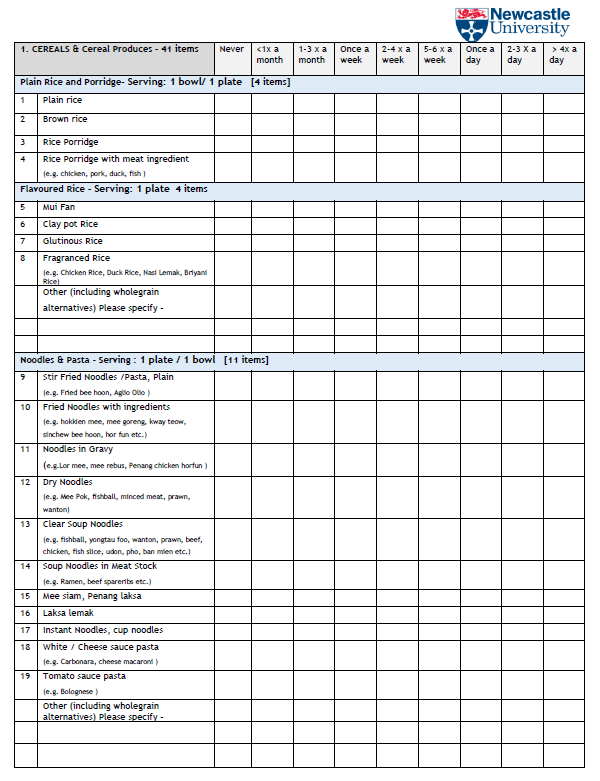

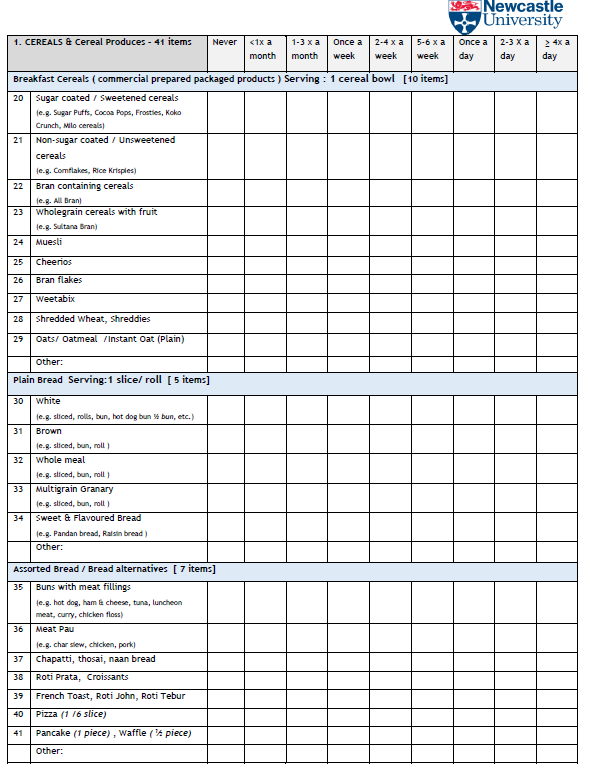


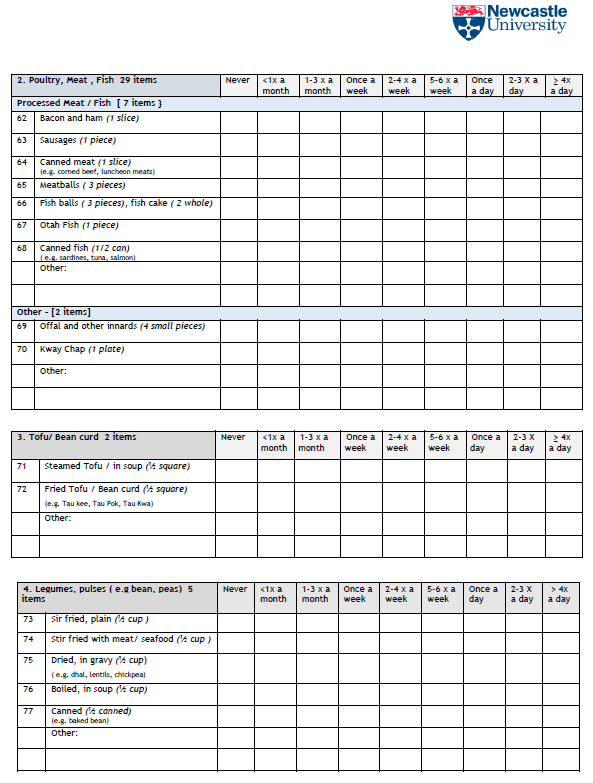
**Appendix 1 Food Frequency Questionnaire for the Mothers (FFQ-M)**


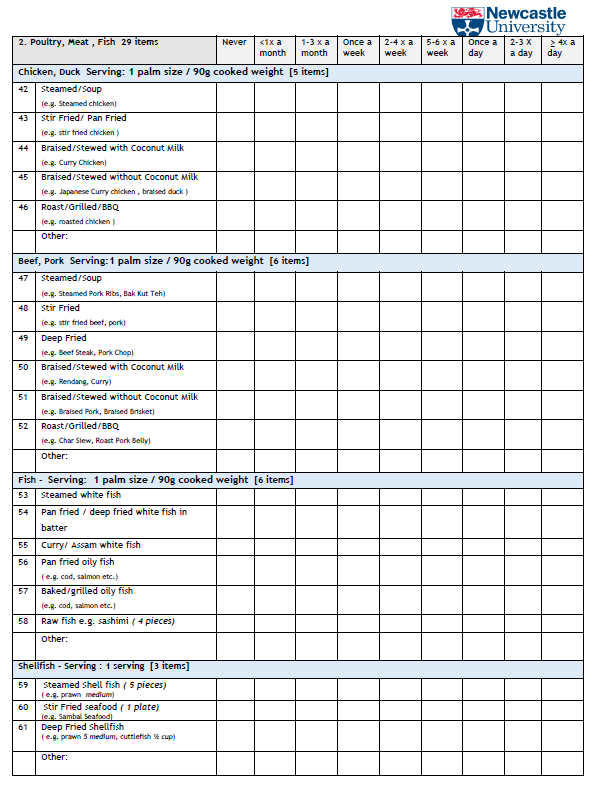


**Appendix 1 Food Frequency Questionnaire for the Mothers (FFQ-M)**


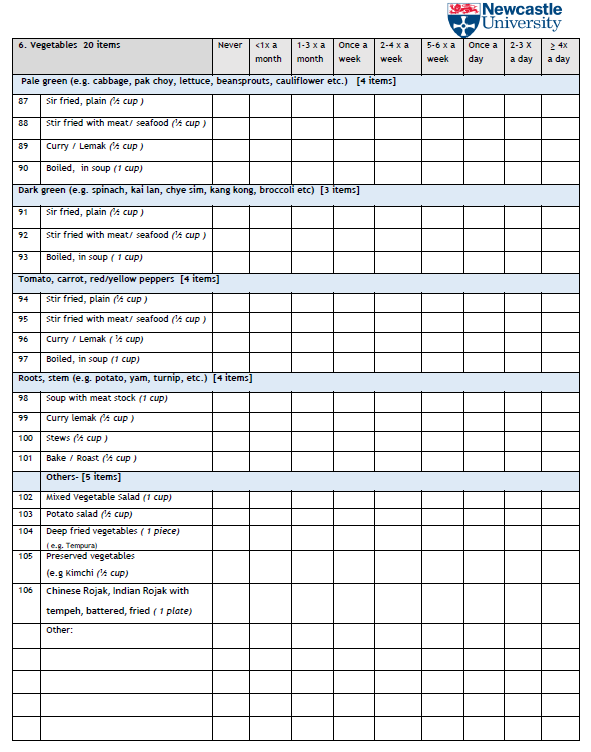


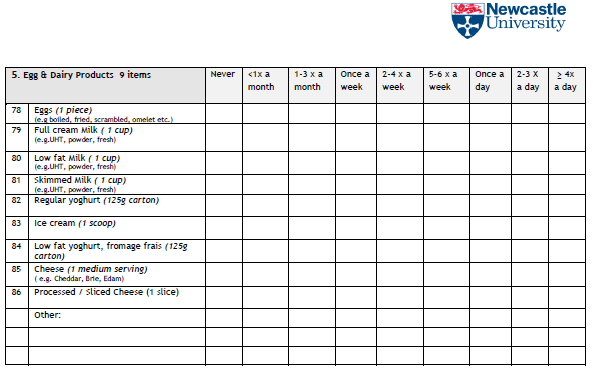


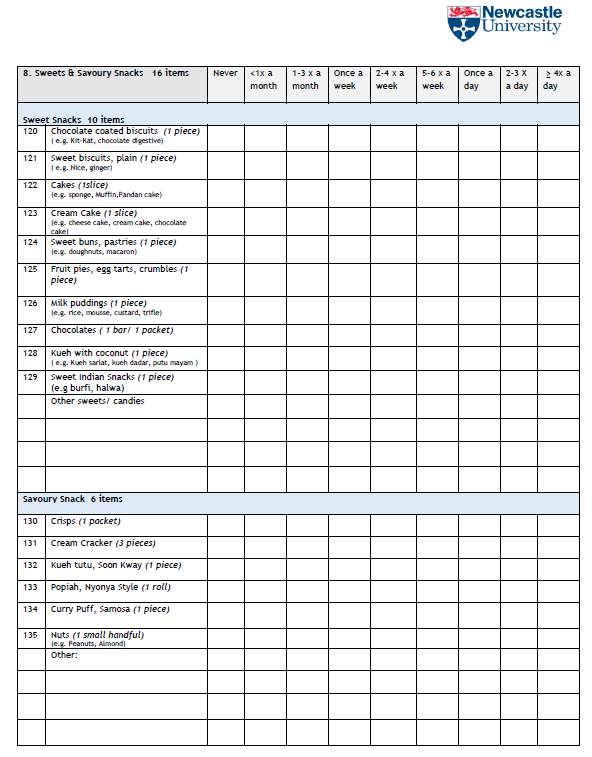
**Appendix 1 Food Frequency Questionnaire for the Mothers (FFQ-M)**


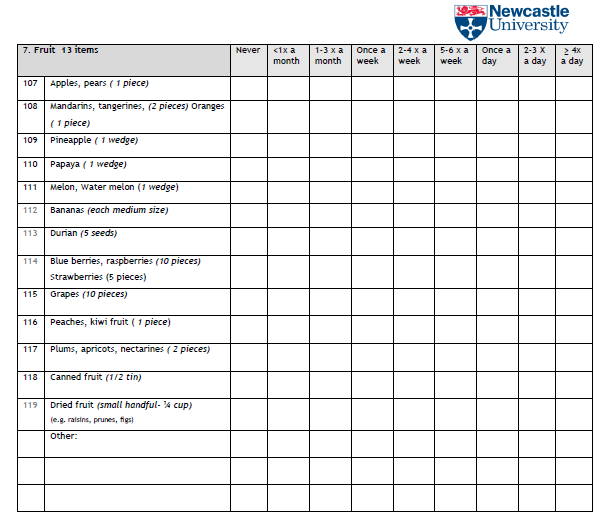


**Appendix 1 Food Frequency Questionnaire for the Mothers (FFQ-M)**


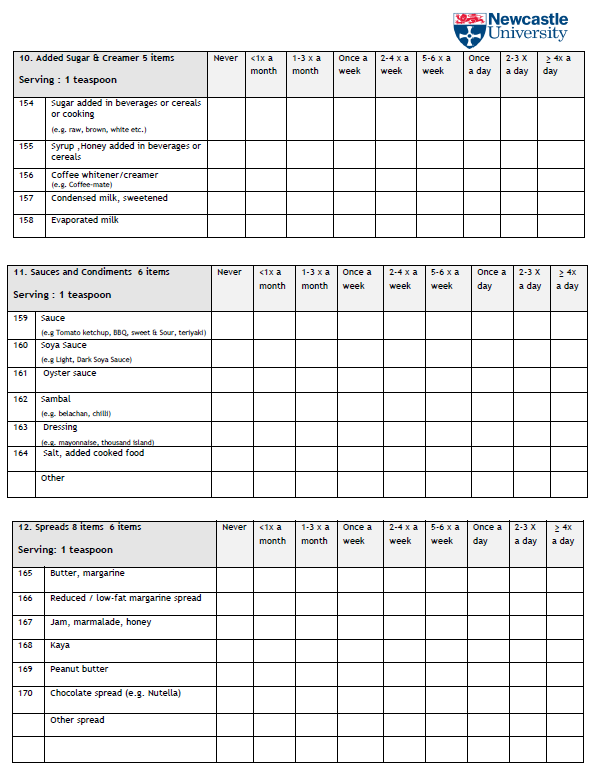


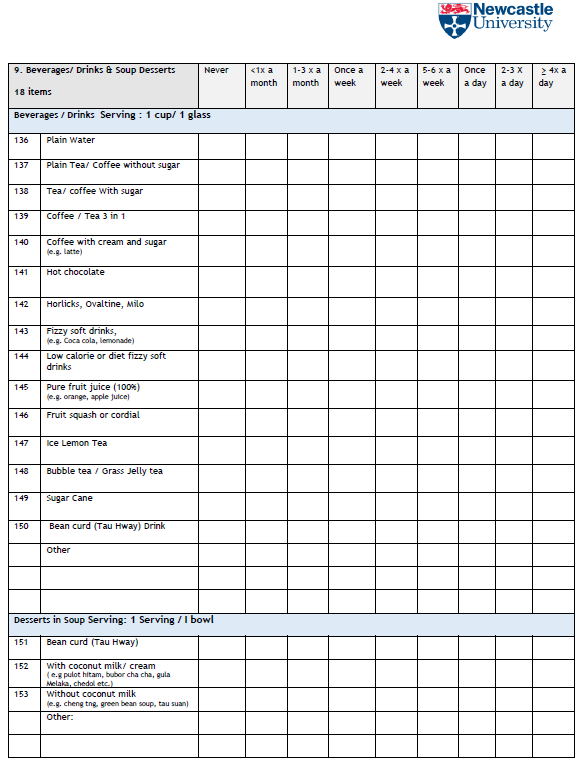


**Appendix 1 Food Frequency Questionnaire for the Mothers (FFQ-M)**


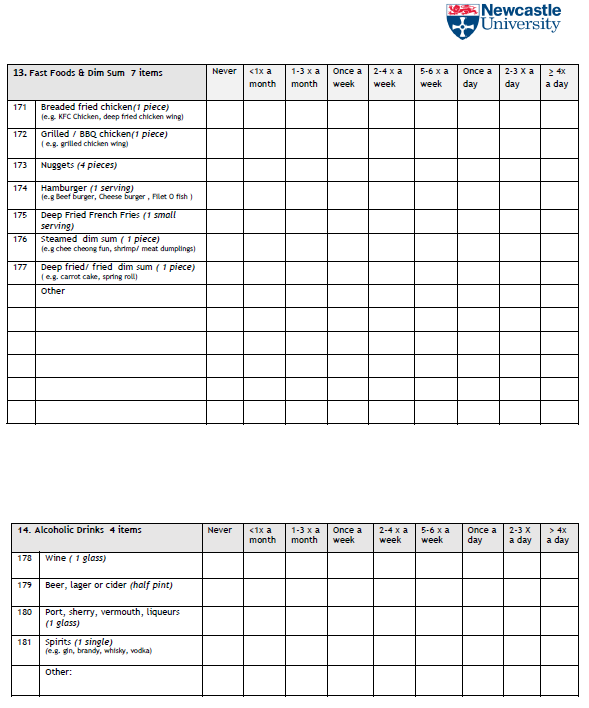


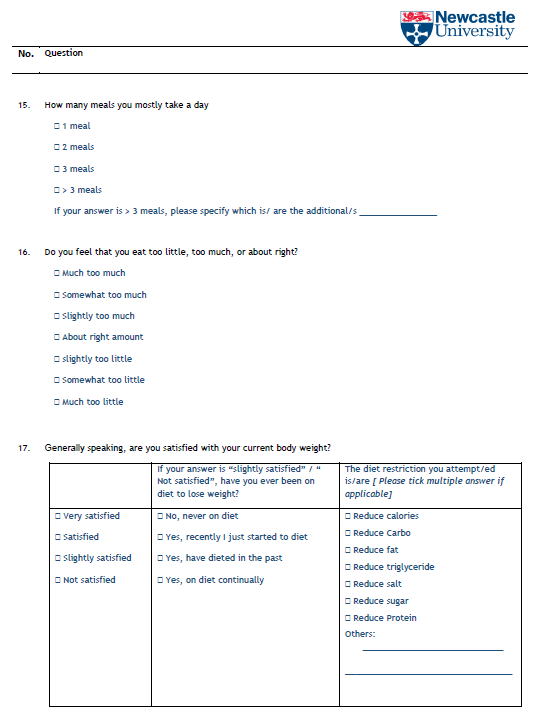


**Appendix 1 Food Frequency Questionnaire for the Mothers (FFQ-M)**


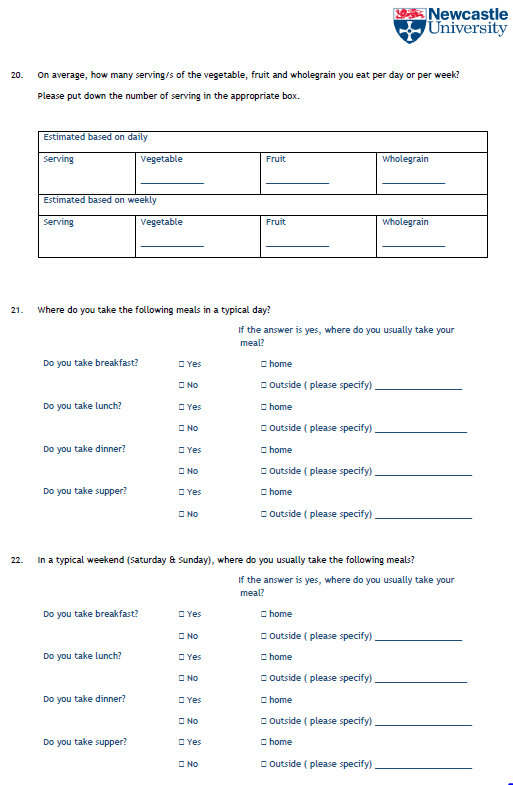

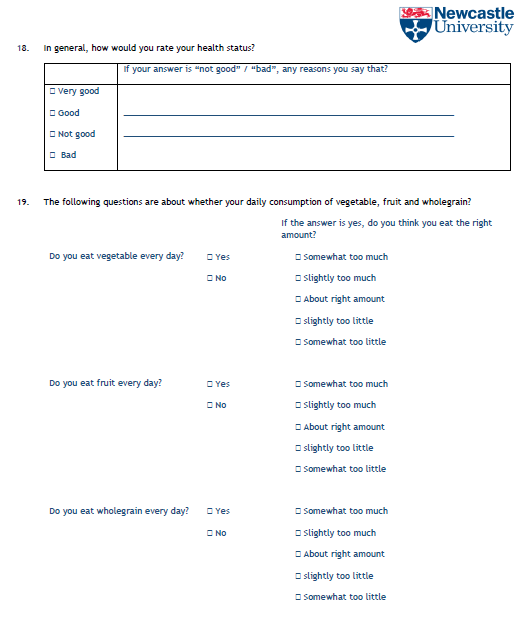


**Appendix 1 Food Frequency Questionnaire for the Mothers (FFQ-M)**


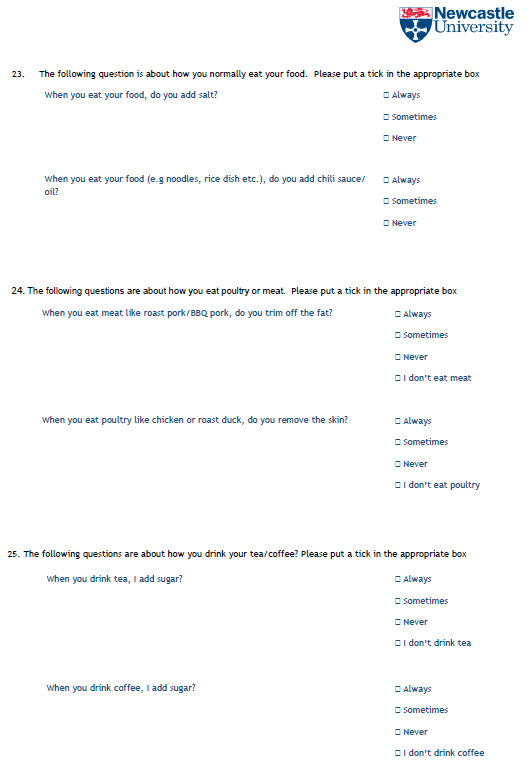

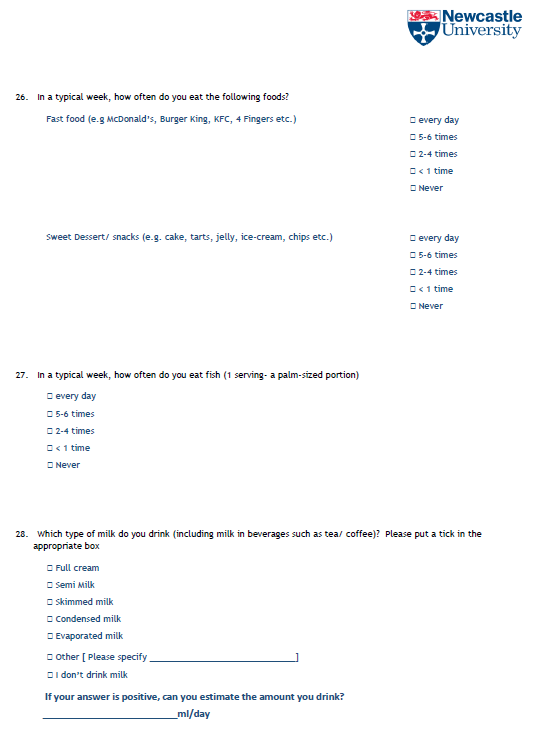


**Appendix 1 Food Frequency Questionnaire for the Mothers (FFQ-M)**


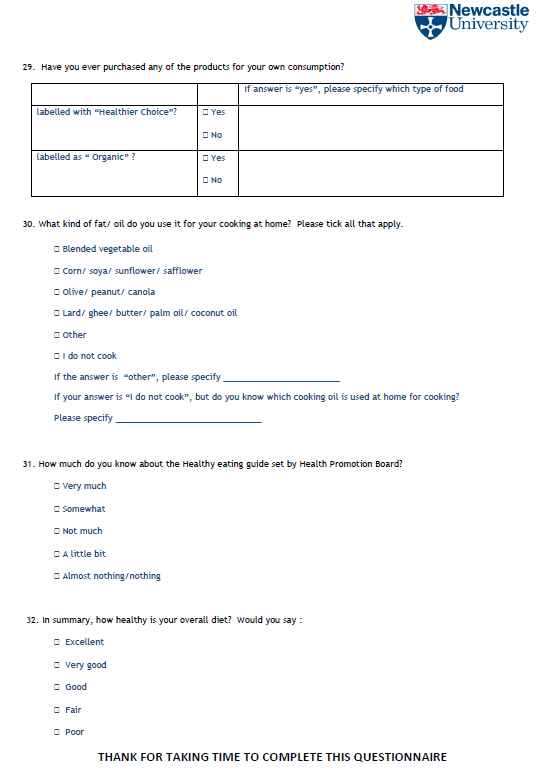


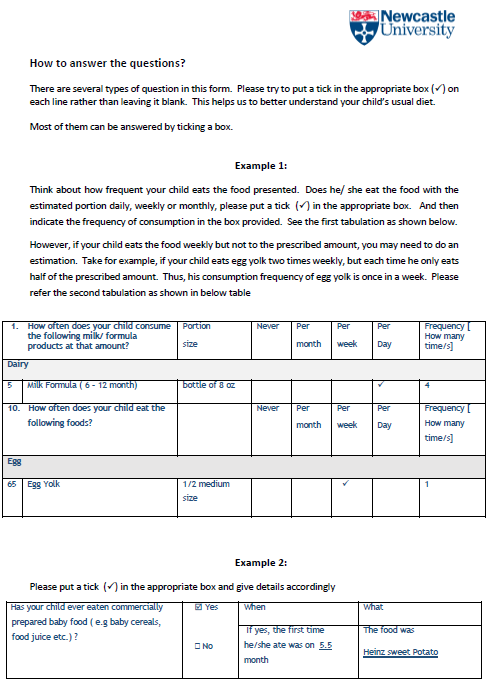
Appendix 2 Food Frequency Questionnaire for the Children (FFQ-C)


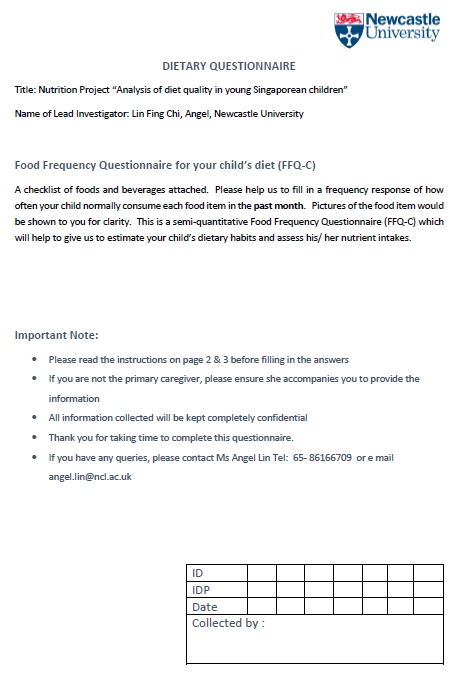


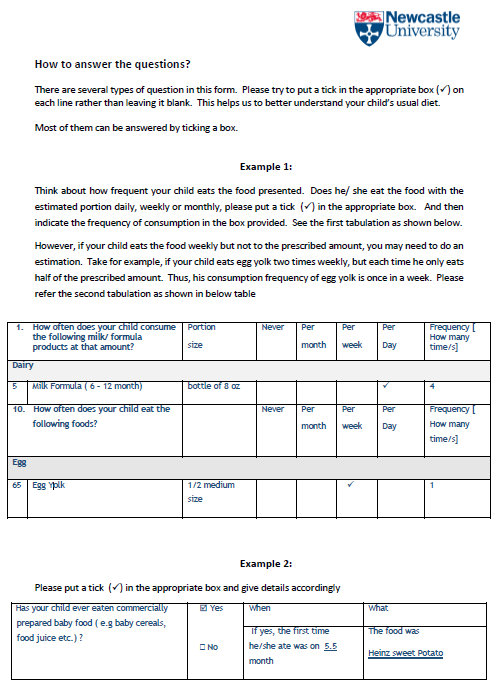
**Appendix 2 Food Frequency Questionnaire for the Children (FFQ-C)**


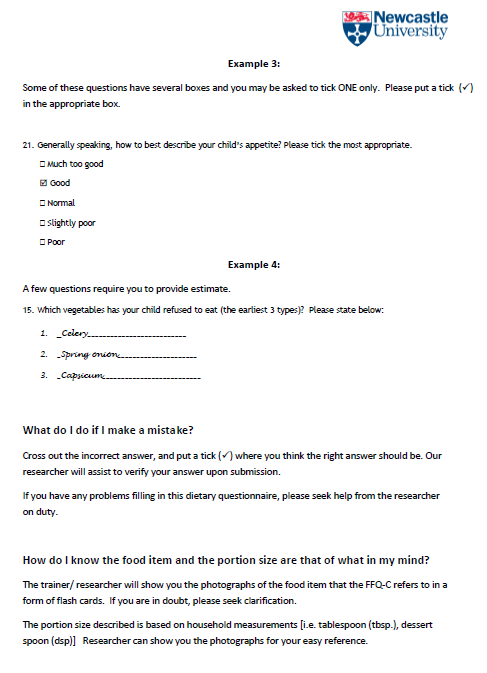


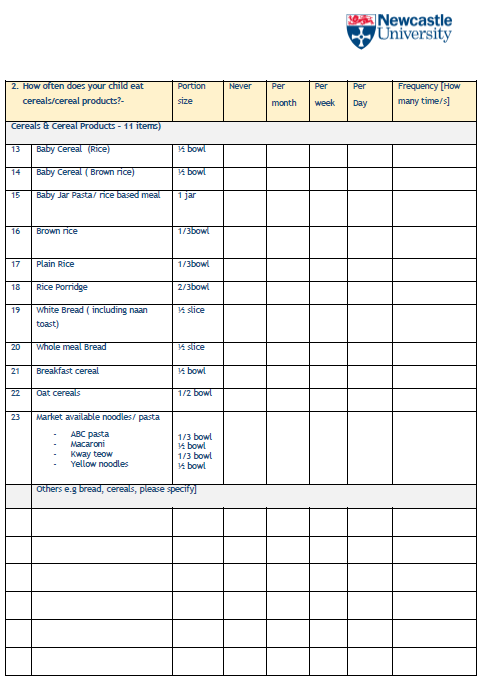
**
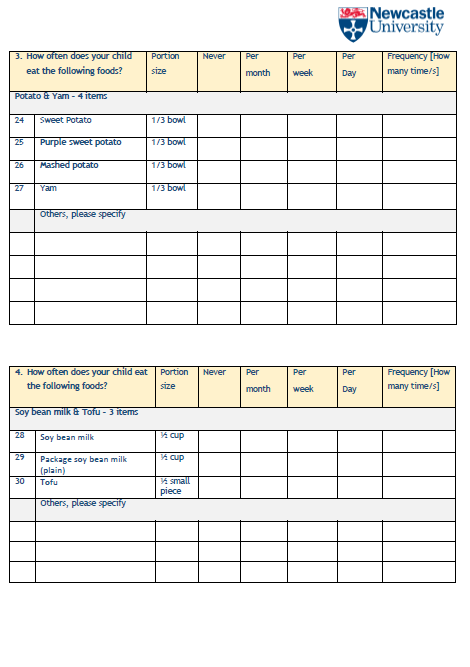
Appendix 2 Food Frequency Questionnaire for the children (FFQ-C)**

**
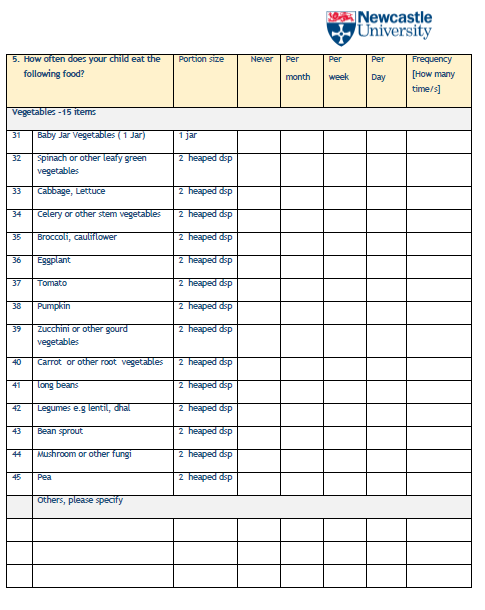
Appendix 2 Food Frequency Questionnaire for the Children (FFQ-C)**
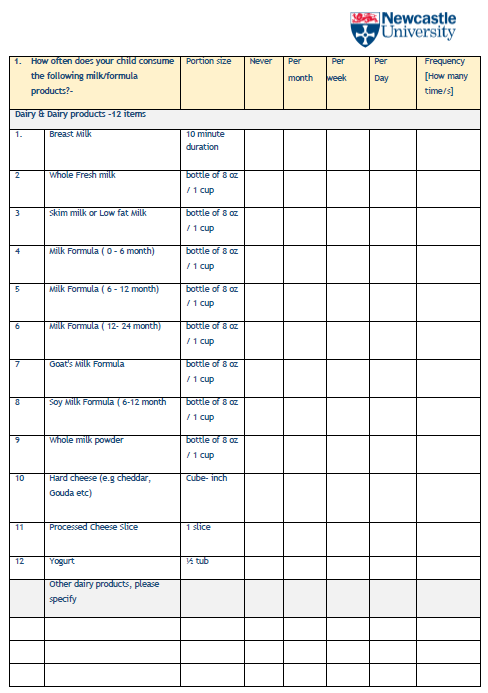


**
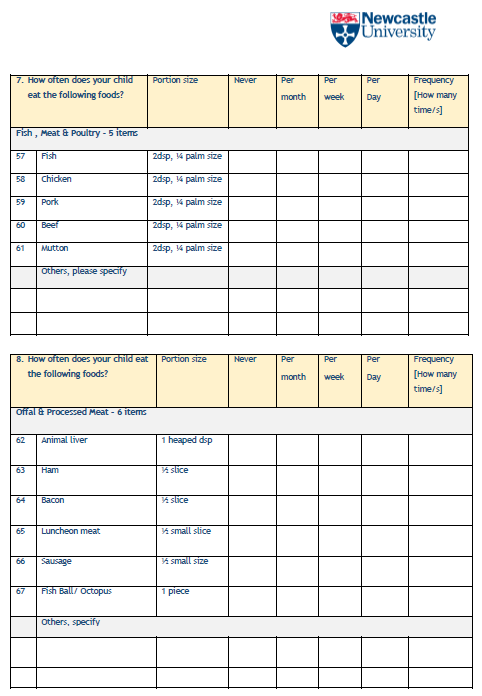

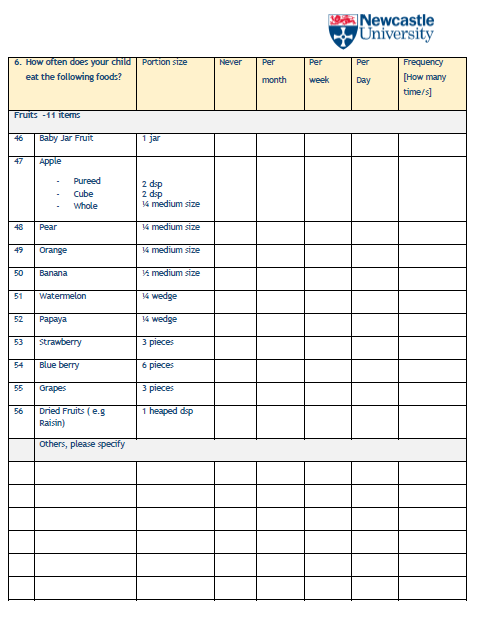
Appendix 2 Food Frequency Questionnaire for the Children (FFQ-C)**

**Appendix 2 Food Frequency Questionnaire for the Children (FFQ-C)**

**
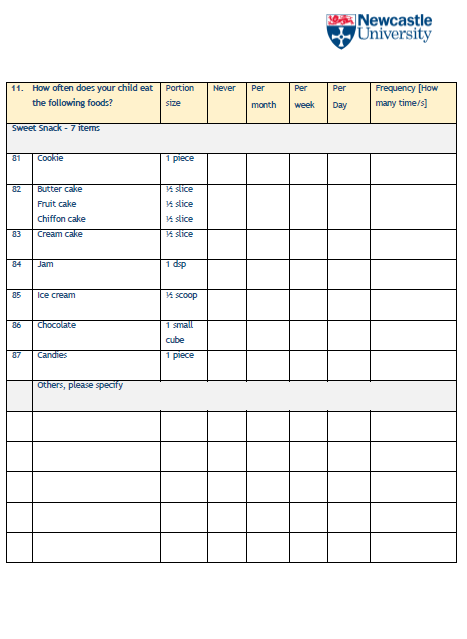

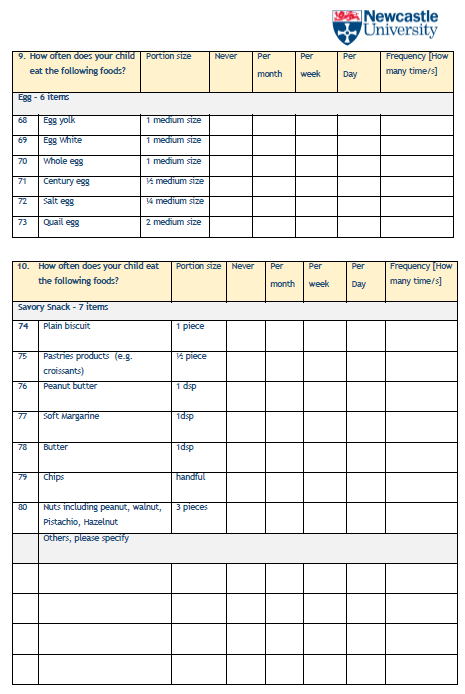
**

**Appendix 2 Food Frequency Questionnaire for the Children (FFQ-C)**


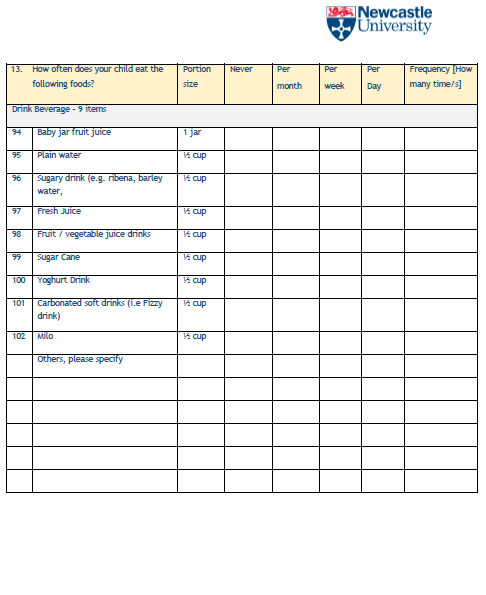

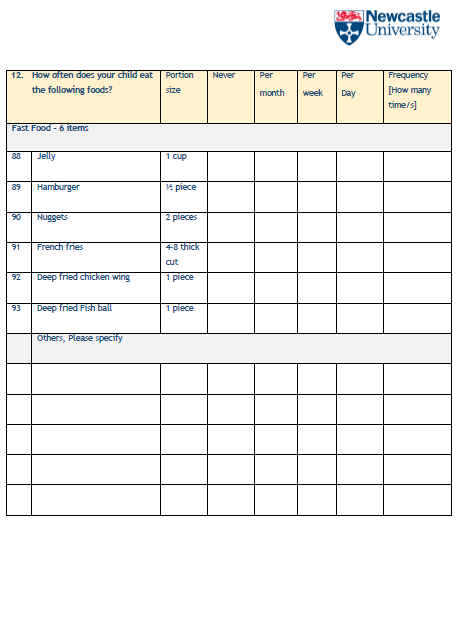


**Appendix 2 Food Frequency Questionnaire for the Children (FFQ-C)**


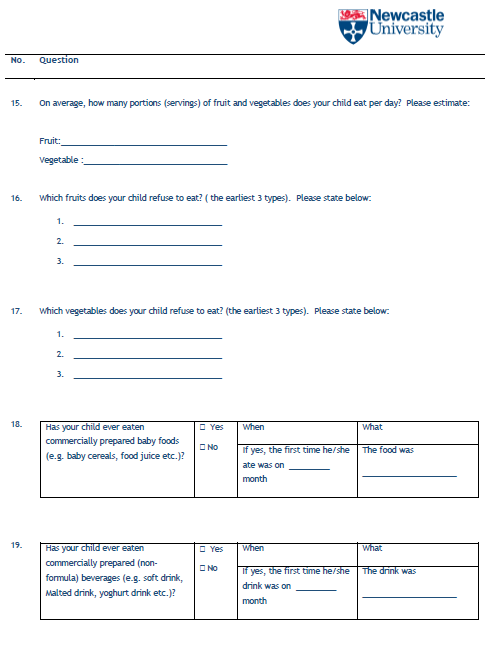

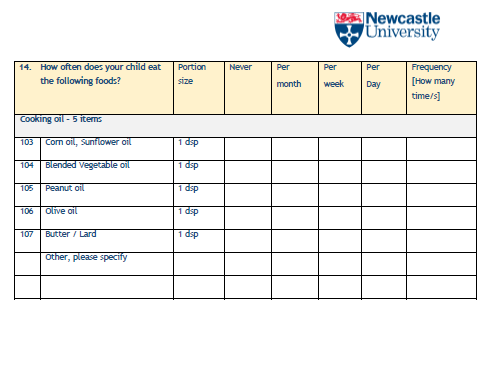


**Appendix 2 Food Frequency Questionnaire for the Children (FFQ-C)**


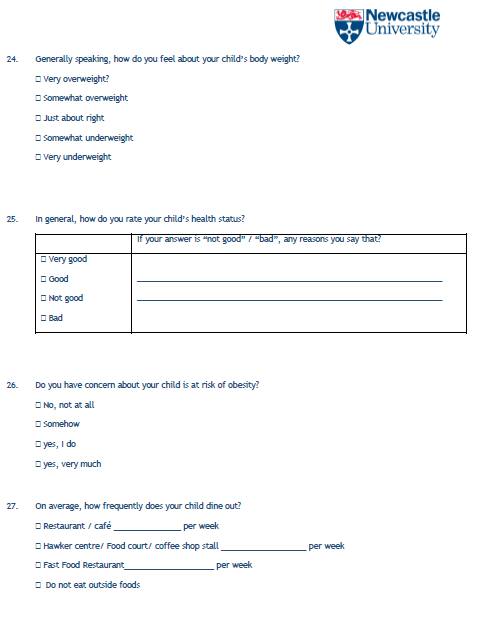

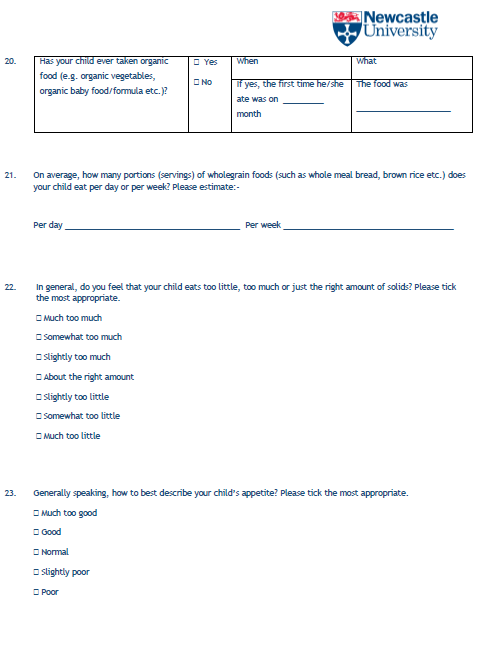


**Appendix 2 Food Frequency Questionnaire for the Children (FFQ-C)**


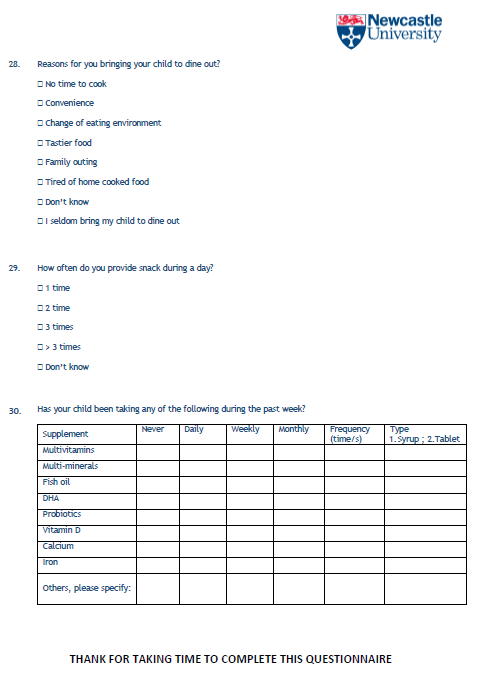


**Appendix 3a**

|  | Age (month) | Target energy intake (kcal/d) | MAX and MIN scores based on frequency of intake (servings/1000kcal/d) | | | | | | | | | | | | MAX and MIN scores based on intake (mg/1000kcal/d) | | MAX and MIN scores based on % daily energy intake | | | | | | |
| --- | --- | --- | --- | --- | --- | --- | --- | --- | --- | --- | --- | --- | --- | --- | --- | --- | --- | --- | --- | --- | --- | --- | --- |
|  |  |  | Rice and Alt MAX score if ≥ | Rice and Alt MIN score if = | Whole grains MAX score if ≥ | Whole grains MIN score if = | Fruit MAX score if ≥ | Fruit MIN score if = | Veget-ables  MAX score if ≥ | Veget-ables  MIN score if = | Meat and Alt MAX score if ≥ | Meat and Alt MIN score if = | Dairy and Alt MAX score if ≥ | Dairy and Alt MIN score if = | Sodium MAX score if ≤ | Sodium MIN score if ≥ | %E fat MAX score if ≤ | %E fat MIN score if ≥ | %E satfat MAX score if ≤ | %E satfat MIN score if ≥ | Added sugar MAX score  if ≤ | | Added sugar MIN score if ≥ |
| Female participants | 6 | 560 | 1.8 | 0.0 | NA | NA | 0.9 | 0.0 | 0.9 | 0.0 | 0.9 | 0.0 | 2.7 | 0.0 | 696.4 | 1742.9 | 30.0 | 40.0 | 10.0 | 20.0 | 5.0 | | 20.0 |
|  | 7 | 570 | 1.8 | 0.0 | NA | NA | 0.9 | 0.0 | 0.9 | 0.0 | 0.9 | 0.0 | 2.6 | 0.0 | 684.2 | 1712.3 | 30.0 | 40.0 | 10.0 | 20.0 | 5.0 | | 20.0 |
|  | 8 | 590 | 1.7 | 0.0 | NA | NA | 0.8 | 0.0 | 0.8 | 0.0 | 0.8 | 0.0 | 2.5 | 0.0 | 661.0 | 1654.2 | 30.0 | 40.0 | 10.0 | 20.0 | 5.0 | | 20.0 |
|  | 9 | 620 | 1.6 | 0.0 | NA | NA | 0.8 | 0.0 | 0.8 | 0.0 | 0.8 | 0.0 | 2.4 | 0.0 | 629.0 | 1574.2 | 30.0 | 40.0 | 10.0 | 20.0 | 5.0 | | 20.0 |
|  | 10 | 630 | 1.6 | 0.0 | NA | NA | 0.8 | 0.0 | 0.8 | 0.0 | 0.8 | 0.0 | 2.4 | 0.0 | 619.0 | 1549.2 | 30.0 | 40.0 | 10.0 | 20.0 | 5.0 | | 20.0 |
|  | 11 | 640 | 1.6 | 0.0 | NA | NA | 0.8 | 0.0 | 0.8 | 0.0 | 0.8 | 0.0 | 2.3 | 0.0 | 609.4 | 1525.0 | 30.0 | 40.0 | 10.0 | 20.0 | 5.0 | | 20.0 |
|  | 12 | 640 | 1.6 | 0.0 | NA | NA | 0.8 | 0.0 | 0.8 | 0.0 | 0.8 | 0.0 | 2.3 | 0.0 | 609.4 | 1525.0 | 30.0 | 40.0 | 10.0 | 20.0 | 5.0 | | 20.0 |
|  | ≥12-≤24 | 810 | 2.5 | 0.0 | 1.2 | 0.0 | 0.6 | 0.0 | 0.6 | 0.0 | 0.6 | 0.0 | 1.9 | 0.0 | 1203.7 | 1234.6 | 30.0 | 40.0 | 10.0 | 20.0 | 5.0 | | 20.0 |
|  | 24- | 1000 | 2.0 | 0.0 | 1.0 | 0.0 | 0.5 | 0.0 | 0.5 | 0.0 | 0.5 | 0.0 | 1.5 | 0.0 | 975.0 | 1000.0 | 30.0 | 40.0 | 10.0 | 20.0 | 5.0 | | 20.0 |
| Male participants | 6 | 600 | 1.7 | 0.0 | NA | NA | 0.8 | 0.0 | 0.8 | 0.0 | 0.8 | 0.0 | 2.5 | 0.0 | 650.0 | 1625.0 | 30.0 | 40.0 | 10.0 | 20.0 | 5.0 | | 20.0 |
|  | 7 | 620 | 1.6 | 0.0 | NA | NA | 0.8 | 0.0 | 0.8 | 0.0 | 0.8 | 0.0 | 2.4 | 0.0 | 629.0 | 1572.6 | 30.0 | 40.0 | 10.0 | 20.0 | 5.0 | | 20.0 |
|  | 8 | 650 | 1.5 | 0.0 | NA | NA | 0.8 | 0.0 | 0.8 | 0.0 | 0.8 | 0.0 | 2.3 | 0.0 | 600.0 | 1500.0 | 30.0 | 40.0 | 10.0 | 20.0 | 5.0 | | 20.0 |
|  | 9 | 670 | 1.5 | 0.0 | NA | NA | 0.7 | 0.0 | 0.7 | 0.0 | 0.7 | 0.0 | 2.2 | 0.0 | 582.1 | 1455.2 | 30.0 | 40.0 | 10.0 | 20.0 | 5.0 | | 20.0 |
|  | 10 | 690 | 1.4 | 0.0 | NA | NA | 0.7 | 0.0 | 0.7 | 0.0 | 0.7 | 0.0 | 2.2 | 0.0 | 565.2 | 1413.0 | 30.0 | 40.0 | 10.0 | 20.0 | 5.0 | | 20.0 |
|  | 11 | 680 | 1.5 | 0.0 | NA | NA | 0.7 | 0.0 | 0.7 | 0.0 | 0.7 | 0.0 | 2.2 | 0.0 | 573.5 | 1433.8 | 30.0 | 40.0 | 10.0 | 20.0 | 5.0 | | 20.0 |
|  | 12 | 740 | 1.4 | 0.0 | NA | NA | 0.7 | 0.0 | 0.7 | 0.0 | 0.7 | 0.0 | 2.0 | 0.0 | 527.0 | 1317.6 | 30.0 | 40.0 | 10.0 | 20.0 | 5.0 | | 20.0 |
|  | ≥12-≤24 | 880 | 2.3 | 0.0 | 1.1 | 0.0 | 0.6 | 0.0 | 0.6 | 0.0 | 0.6 | 0.0 | 1.7 | 0.0 | 1108.0 | 1136.4 | 30.0 | 40.0 | 10.0 | 20.0 | 5.0 | | 20.0 |
|  | 24- | 1080 | 1.9 | 0.0 | 0.9 | 0.0 | 0.5 | 0.0 | 0.5 | 0.0 | 0.5 | 0.0 | 1.4 | 0.0 | 902.8 | 925.9 | 30.0 | 40.0 | 10.0 | 20.0 | 5.0 | | 20.0 |
| Alt: Alternative; %E: Percentage of energy; sat fat: saturated fat | | | | | | | | | | | | | | | | | | | | |  |  | |

The recommended dietary intake of energy differs by age, sex, physical activity levels and stages of pregnancy. The serving recommendations for food and nutrient were energy adjusted and expressed per 1000Kcal.

| **Appendix 3b (i)** | | | | | | | | | | | | | | | | | | | | | | |  |
| --- | --- | --- | --- | --- | --- | --- | --- | --- | --- | --- | --- | --- | --- | --- | --- | --- | --- | --- | --- | --- | --- | --- | --- |
| **Age (y)** | | **Physical activity level** | Target energy intake (kcal/d) | MAX and MIN scores based on frequency of intake (servings/1000kcal/d) | | | | | | | | | | | | MAX and MIN scores based on intake (mg/1000kcal/d) | | MAX and MIN scores based on % daily energy intake | | | | | |
|  |  |  |  | Rice and Alt  MAX score if ≥ | Rice and Alt  MIN score if = | Whole grains MAX score if ≥ | Whole grains MIN score if = | Fruit MAX score if ≥ | Fruit  MIN score if = | Veget-ables  MAX  score if ≥ | Veget-ables  MIN  score if = | Meat and Alt MAX score if ≥ | Meat and Alt  MIN score if = | Dairy and Alt  MAX score if ≥ | Dairy and Alt  MIN score if = | Sodium MAX score if ≤ | Sodium MIN score if ≥ | %E fat MAX score if ≤ | %E fat MIN score if ≥ | %E sat fat MAX score if ≤ | %E sat fat MIN score if ≥ | Added sugar MAX score if ≤ | Added sugar MIN score if ≥ |
| 18-29 | | Light | 1745 | 2.9 | 0.0 | 1.1 | 0.0 | 1.1 | 0.0 | 1.1 | 0.0 | 1.1 | 0.0 | 1.1 | 0.0 | 573.1 | 1146.1 | 30.0 | 40.0 | 10.0 | 20.0 | 10.0 | 20.0 |
| 18-29 | | Moderate | 2070 | 2.4 | 0.0 | 1.0 | 0.0 | 1.0 | 0.0 | 1.0 | 0.0 | 1.0 | 0.0 | 1.0 | 0.0 | 483.1 | 966.2 | 30.0 | 40.0 | 10.0 | 20.0 | 10.0 | 20.0 |
| 18-29 | | Vigorous | 2840 | 1.8 | 0.0 | 0.7 | 0.0 | 0.7 | 0.0 | 0.7 | 0.0 | 0.7 | 0.0 | 0.7 | 0.0 | 352.1 | 704.2 | 30.0 | 40.0 | 10.0 | 20.0 | 10.0 | 20.0 |
| 18-29 Lactating | | Light | 2245 | 2.2 | 0.0 | 0.9 | 0.0 | 0.9 | 0.0 | 0.9 | 0.0 | 0.9 | 0.0 | 0.9 | 0.0 | 445.4 | 890.9 | 30.0 | 40.0 | 10.0 | 20.0 | 10.0 | 20.0 |
| 18-29 Lactating | | Moderate | 2570 | 1.9 | 0.0 | 0.8 | 0.0 | 0.8 | 0.0 | 0.8 | 0.0 | 0.8 | 0.0 | 0.8 | 0.0 | 389.1 | 778.2 | 30.0 | 40.0 | 10.0 | 20.0 | 10.0 | 20.0 |
| 18-29 Lactating | | Vigorous | 3340 | 1.5 | 0.0 | 0.6 | 0.0 | 0.6 | 0.0 | 0.6 | 0.0 | 0.6 | 0.0 | 0.6 | 0.0 | 299.4 | 598.8 | 30.0 | 40.0 | 10.0 | 20.0 | 10.0 | 20.0 |
| 18-29 Pregnant 2nd trimester | | Light | 2115 | 2.4 | 0.0 | 0.9 | 0.0 | 0.9 | 0.0 | 0.9 | 0.0 | 0.9 | 0.0 | 0.9 | 0.0 | 472.8 | 945.6 | 30.0 | 40.0 | 10.0 | 20.0 | 10.0 | 20.0 |
| 18-29 Pregnant 2nd trimester | | Moderate | 2440 | 2.0 | 0.0 | 0.8 | 0.0 | 0.8 | 0.0 | 0.8 | 0.0 | 0.8 | 0.0 | 0.8 | 0.0 | 409.8 | 819.7 | 30.0 | 40.0 | 10.0 | 20.0 | 10.0 | 20.0 |
| 18-29 Pregnant 2nd trimester | | Vigorous | 3210 | 1.6 | 0.0 | 0.6 | 0.0 | 0.6 | 0.0 | 0.6 | 0.0 | 0.6 | 0.0 | 0.6 | 0.0 | 311.5 | 623.1 | 30.0 | 40.0 | 10.0 | 20.0 | 10.0 | 20.0 |
| 18-29 Pregnant 3rd trimester | | Light | 2225 | 2.2 | 0.0 | 0.9 | 0.0 | 0.9 | 0.0 | 0.9 | 0.0 | 0.9 | 0.0 | 0.9 | 0.0 | 449.4 | 898.9 | 30.0 | 40.0 | 10.0 | 20.0 | 10.0 | 20.0 |
| 18-29 Pregnant 3rd trimester | | Moderate | 2550 | 2.0 | 0.0 | 0.8 | 0.0 | 0.8 | 0.0 | 0.8 | 0.0 | 0.8 | 0.0 | 0.8 | 0.0 | 392.2 | 784.3 | 30.0 | 40.0 | 10.0 | 20.0 | 10.0 | 20.0 |
| 18-29 Pregnant 3rd trimester | | Vigorous | 3320 | 1.5 | 0.0 | 0.6 | 0.0 | 0.6 | 0.0 | 0.6 | 0.0 | 0.6 | 0.0 | 0.6 | 0.0 | 301.2 | 602.4 | 30.0 | 40.0 | 10.0 | 20.0 | 10.0 | 20.0 |
| 18-29 Pregnant 2nd trimester Lactating | | Light | 2615 | 1.9 | 0.0 | 0.8 | 0.0 | 0.8 | 0.0 | 0.8 | 0.0 | 0.8 | 0.0 | 0.8 | 0.0 | 382.4 | 764.8 | 30.0 | 40.0 | 10.0 | 20.0 | 10.0 | 20.0 |
| 18-29 Pregnant 2nd trimester Lactating | | Moderate | 2940 | 1.7 | 0.0 | 0.7 | 0.0 | 0.7 | 0.0 | 0.7 | 0.0 | 0.7 | 0.0 | 0.7 | 0.0 | 340.1 | 680.3 | 30.0 | 40.0 | 10.0 | 20.0 | 10.0 | 20.0 |
| 18-29 Pregnant 2nd trimester Lactating | | Vigorous | 3710 | 1.3 | 0.0 | 0.5 | 0.0 | 0.5 | 0.0 | 0.5 | 0.0 | 0.5 | 0.0 | 0.5 | 0.0 | 269.5 | 539.1 | 30.0 | 40.0 | 10.0 | 20.0 | 10.0 | 20.0 |
| 18-29 Pregnant 3rd trimester Lactating | | Light | 2725 | 1.8 | 0.0 | 0.7 | 0.0 | 0.7 | 0.0 | 0.7 | 0.0 | 0.7 | 0.0 | 0.7 | 0.0 | 367.0 | 733.9 | 30.0 | 40.0 | 10.0 | 20.0 | 10.0 | 20.0 |
| 18-29 Pregnant 3rd trimester Lactating | | Moderate | 3050 | 1.6 | 0.0 | 0.7 | 0.0 | 0.7 | 0.0 | 0.7 | 0.0 | 0.7 | 0.0 | 0.7 | 0.0 | 327.9 | 655.7 | 30.0 | 40.0 | 10.0 | 20.0 | 10.0 | 20.0 |
| 18-29 Pregnant 3rd trimester Lactating | | Vigorous | 3820 | 1.3 | 0.0 | 0.5 | 0.0 | 0.5 | 0.0 | 0.5 | 0.0 | 0.5 | 0.0 | 0.5 | 0.0 | 261.8 | 523.6 | 30.0 | 40.0 | 10.0 | 20.0 | 10.0 | 20.0 |

| Alt: Alternative; %E: Percentage of energy; sat fat: saturated fat |  |  |
| --- | --- | --- |

The recommended dietary intake of energy differs by age, sex, physical activity levels and stages of pregnancy. The serving recommendations for food and nutrient were energy adjusted and expressed per 1000Kcal.

Alt: Alternative; %E: Percentage of energy; sat fat: saturated fat

The recommended dietary intake of energy differs by age, sex, physical activity levels and stages of pregnancy. The serving recommendations for food and nutrient were energy adjusted and expressed per 1000Kcal.

| **Appendix 3b(ii)** | | | | | | | | | | | | | | | | | | | | | | |
| --- | --- | --- | --- | --- | --- | --- | --- | --- | --- | --- | --- | --- | --- | --- | --- | --- | --- | --- | --- | --- | --- | --- |
| **Age (y)** | Physical activity level | Target energy intake (kcal/d) | MAX and MIN scores based on frequency of intake (servings/1000kcal/d) | | | | | | | | | | | | MAX and MIN scores based on intake (mg/1000kcal/d) | | MAX and MIN scores based on % daily energy intake | | | | | |
|  |  |  | Rice and Alt  MAX score if ≥ | Rice and Alt  MIN score if = | Whole grains MAX score if ≥ | Whole grains MIN score if = | Fruit MAX score if ≥ | Fruit  MIN score if = | Veget-ables  MAX  score if ≥ | Veget-  ables  MIN  score if = | Meat and Alt MAX score if ≥ | Meat and Alt  MIN score if = | Dairy and Alt  MAX score if ≥ | Dairy and Alt  MIN score if = | Sodium MAX score if ≤ | Sodium MIN score if ≥ | %E fat MAX score if ≤ | %E fat MIN score if ≥ | %E sat fat MAX score if ≤ | %E sat fat MIN score if ≥ | Added sugar MAX score if ≤ | Added sugar MIN score if ≥ |
| 30-59 | Light | 1720 | 2.9 | 0.0 | 1.2 | 0.0 | 1.2 | 0.0 | 1.2 | 0.0 | 1.2 | 0.0 | 1.2 | 0.0 | 581.4 | 1162.8 | 30.0 | 40.0 | 10.0 | 20.0 | 10.0 | 20.0 |
| 30-59 | Moderate | 2035 | 2.5 | 0.0 | 1.0 | 0.0 | 1.0 | 0.0 | 1.0 | 0.0 | 1.0 | 0.0 | 1.0 | 0.0 | 491.4 | 982.8 | 30.0 | 40.0 | 10.0 | 20.0 | 10.0 | 20.0 |
| 30-59 | Vigorous | 2800 | 1.8 | 0.0 | 0.7 | 0.0 | 0.7 | 0.0 | 0.7 | 0.0 | 0.7 | 0.0 | 0.7 | 0.0 | 357.1 | 714.3 | 30.0 | 40.0 | 10.0 | 20.0 | 10.0 | 20.0 |
| 30-59 Lactating | Light | 2220 | 2.3 | 0.0 | 0.9 | 0.0 | 0.9 | 0.0 | 0.9 | 0.0 | 0.9 | 0.0 | 0.9 | 0.0 | 450.5 | 900.9 | 30.0 | 40.0 | 10.0 | 20.0 | 10.0 | 20.0 |
| 30-59 Lactating | Moderate | 2535 | 2.0 | 0.0 | 0.8 | 0.0 | 0.8 | 0.0 | 0.8 | 0.0 | 0.8 | 0.0 | 0.8 | 0.0 | 394.5 | 789.0 | 30.0 | 40.0 | 10.0 | 20.0 | 10.0 | 20.0 |
| 30-59 Lactating | Vigorous | 3300 | 1.5 | 0.0 | 0.6 | 0.0 | 0.6 | 0.0 | 0.6 | 0.0 | 0.6 | 0.0 | 0.6 | 0.0 | 303.0 | 606.1 | 30.0 | 40.0 | 10.0 | 20.0 | 10.0 | 20.0 |
| 30-59 Pregnant 2nd trimester | Light | 2090 | 2.4 | 0.0 | 1.0 | 0.0 | 1.0 | 0.0 | 1.0 | 0.0 | 1.0 | 0.0 | 1.0 | 0.0 | 478.5 | 956.9 | 30.0 | 40.0 | 10.0 | 20.0 | 10.0 | 20.0 |
| 30-59 Pregnant 2nd trimester | Moderate | 2405 | 2.1 | 0.0 | 0.8 | 0.0 | 0.8 | 0.0 | 0.8 | 0.0 | 0.8 | 0.0 | 0.8 | 0.0 | 415.8 | 831.6 | 30.0 | 40.0 | 10.0 | 20.0 | 10.0 | 20.0 |
| 30-59 Pregnant 2nd trimester | Vigorous | 3170 | 1.6 | 0.0 | 0.6 | 0.0 | 0.6 | 0.0 | 0.6 | 0.0 | 0.6 | 0.0 | 0.6 | 0.0 | 315.5 | 630.9 | 30.0 | 40.0 | 10.0 | 20.0 | 10.0 | 20.0 |
| 30-59 Pregnant 3rd trimester | Light | 2700 | 1.9 | 0.0 | 0.7 | 0.0 | 0.7 | 0.0 | 0.7 | 0.0 | 0.7 | 0.0 | 0.7 | 0.0 | 370.4 | 740.7 | 30.0 | 40.0 | 10.0 | 20.0 | 10.0 | 20.0 |
| 30-59 Pregnant 3rd trimester | Moderate | 3015 | 1.7 | 0.0 | 0.7 | 0.0 | 0.7 | 0.0 | 0.7 | 0.0 | 0.7 | 0.0 | 0.7 | 0.0 | 331.7 | 663.3 | 30.0 | 40.0 | 10.0 | 20.0 | 10.0 | 20.0 |
| 30-59 Pregnant 3rd trimester | Vigorous | 3780 | 1.3 | 0.0 | 0.5 | 0.0 | 0.5 | 0.0 | 0.5 | 0.0 | 0.5 | 0.0 | 0.5 | 0.0 | 264.6 | 529.1 | 30.0 | 40.0 | 10.0 | 20.0 | 10.0 | 20.0 |
| 30-59 Pregnant 2nd trimester Lactating | Light | 2590 | 1.9 | 0.0 | 0.8 | 0.0 | 0.8 | 0.0 | 0.8 | 0.0 | 0.8 | 0.0 | 0.8 | 0.0 | 386.1 | 772.2 | 30.0 | 40.0 | 10.0 | 20.0 | 10.0 | 20.0 |
| 30-59 Pregnant 2nd trimester Lactating | Moderate | 2905 | 1.7 | 0.0 | 0.7 | 0.0 | 0.7 | 0.0 | 0.7 | 0.0 | 0.7 | 0.0 | 0.7 | 0.0 | 344.2 | 688.5 | 30.0 | 40.0 | 10.0 | 20.0 | 10.0 | 20.0 |
| 30-59 Pregnant 2nd trimester Lactating | Vigorous | 3670 | 1.4 | 0.0 | 0.5 | 0.0 | 0.5 | 0.0 | 0.5 | 0.0 | 0.5 | 0.0 | 0.5 | 0.0 | 272.5 | 545.0 | 30.0 | 40.0 | 10.0 | 20.0 | 10.0 | 20.0 |
| 30-59 Pregnant 3rd trimester Lactating | Light | 3200 | 1.6 | 0.0 | 0.6 | 0.0 | 0.6 | 0.0 | 0.6 | 0.0 | 0.6 | 0.0 | 0.6 | 0.0 | 312.5 | 625.0 | 30.0 | 40.0 | 10.0 | 20.0 | 10.0 | 20.0 |
| 30-59 Pregnant 3rd trimester Lactating | Moderate | 3515 | 1.4 | 0.0 | 0.6 | 0.0 | 0.6 | 0.0 | 0.6 | 0.0 | 0.6 | 0.0 | 0.6 | 0.0 | 284.5 | 569.0 | 30.0 | 40.0 | 10.0 | 20.0 | 10.0 | 20.0 |
| 30-59 Pregnant 3rd trimester Lactating | Vigorous | 4280 | 1.2 | 0.0 | 0.5 | 0.0 | 0.5 | 0.0 | 0.5 | 0.0 | 0.5 | 0.0 | 0.5 | 0.0 | 233.6 | 467.3 | 30.0 | 40.0 | 10.0 | 20.0 | 10.0 | 20.0 |
